# Treatment of *Porphyromonas gulae* infection and downstream pathology in the aged dog by lysine‐gingipain inhibitor COR388

**DOI:** 10.1002/prp2.562

**Published:** 2020-01-30

**Authors:** Shirin Arastu‐Kapur, Mai Nguyen, Debasish Raha, Florian Ermini, Ursula Haditsch, Joseph Araujo, Ines A. M. De Lannoy, Mark I. Ryder, Stephen S. Dominy, Casey Lynch, Leslie J. Holsinger

**Affiliations:** ^1^ Cortexyme, Inc. South San Francisco CA USA; ^2^ InterVivo Solutions Toronto ON Canada; ^3^ University of California San Francisco San Francisco CA USA

**Keywords:** Alzheimer's disease, gingipain inhibitor, *P. gingivalis*, *P. gulae*, periodontal disease

## Abstract

COR388, a small‐molecule lysine‐gingipain inhibitor, is currently being investigated in a Phase 2/3 clinical trial for Alzheimer's disease (AD) with exploratory endpoints in periodontal disease. Gingipains are produced by two species of bacteria, *Porphyromonas gingivalis* and *Porphyromonas gulae*, typically associated with periodontal disease and systemic infections in humans and dogs, respectively. *P. gulae* infection in dogs is associated with periodontal disease, which provides a physiologically relevant model to investigate the pharmacology of COR388. In the current study, aged dogs with a natural oral infection of *P. gulae* and periodontal disease were treated with COR388 by oral administration for up to 90 days to assess lysine‐gingipain target engagement and reduction of bacterial load and downstream pathology. In a 28‐day dose‐response study, COR388 inhibited the lysine‐gingipain target and reduced *P. gulae* load in saliva, buccal cells, and gingival crevicular fluid. The lowest effective dose was continued for 90 days and was efficacious in continuous reduction of bacterial load and downstream periodontal disease pathology. In a separate histology study, dog brain tissue showed evidence of *P. gulae* DNA and neuronal lysine‐gingipain, demonstrating that *P. gulae* infection is systemic and spreads beyond its oral reservoir, similar to recent observations of *P. gingivalis* in humans. Together, the pharmacokinetics and pharmacodynamics of COR388 lysine‐gingipain inhibition, along with reduction of bacterial load and periodontal disease in naturally occurring *P. gulae* infection in the dog, support the use of COR388 in targeting lysine‐gingipain and eliminating *P. gingivalis* infection in humans.

**Abbreviations**
AD: Alzheimer's disease
CCAC: Canadian Council on Animal Care
GCF: Gingival crevicular fluid
IACUC: Institutional Animal Care and Use Committee
SGP: subgingival plaque

## 1 INTRODUCTION

COR388 is an irreversible active‐site inhibitor developed to target lysine‐gingipain (Kgp) in the brain of Alzheimer's disease (AD) patients.^1^ Kgp is a cysteine protease virulence factor secreted by *Porphyromonas gingivalis*, a keystone bacterium in the development of periodontal disease.^2^ The secretion of gingipain proteases is part of the asaccharolytic metabolism of *P. gingivalis,* and the gingipains are known to contribute to dysbiosis, immune pathway induction and dysregulation, chronic inflammation, and cellular toxicity.^3^, ^4^, ^5^, ^6^, ^7^ While *P. gingivalis* resides in oral biofluids and tissues, it has also been shown to translocate to other tissues where it is associated with disease pathology including atherosclerosis,^8^ cancer,^9^ arthritis,^10^ and AD.^1^, ^11^

*P. gingivalis* is best known for its pathogenic role in periodontal disease, and periodontal disease is a risk factor for the development of AD,^12^, ^13^ indicating that a high bacterial load in the oral cavity may be one of many risk factors, along with age, genetics, bacterial strain virulence, and other variables that may contribute to a chronic brain infection with *P. gingivalis*. Additionally, ongoing moderate to severe periodontal disease has been shown to result in more rapid worsening of cognitive decline in clinical AD patients.^14^ We recently identified gingipains and *P. gingivalis* in the brains of subjects with AD, and brain gingipain levels positively correlated to severity of AD diagnosis and pathology.^1^ Mechanistic studies in wild‐type mice demonstrated that oral infection with *P. gingivalis* results in brain colonization and pathology characteristic of AD,^1^, ^11^ and these effects were blocked or reversed by COR388.^1^

As noted above, we and others have found that oral infection of mice with *P. gingivalis* results in translocation to the brain, resulting in neurodegeneration and AD pathology, while other oral bacteria did not translocate.^15^ Because dogs are naturally infected with the closely related species, *P. gulae,* and commonly develop both periodontal disease^16^ and canine cognitive dysfunction associated with cerebral neuropathology, including amyloidosis, tau hyperphosphorylation and neuronal loss that resembles AD in humans,^17^, ^18^ we aimed to examine the presence of *P. gulae* and associated gingipain proteases in dogs and investigate the pharmacology of the selective Kgp inhibitor COR388 in these animals. The prevalence of periodontal disease in dogs increases with age, with 40.8% of dogs 1‐4 years old, and 53.6% of dogs 5‐8 years old, having evidence of the disease, with prevalence increasing to >85% in dogs older than 8 years of age.^19^ It was originally thought that *P. gulae* was the animal biotype of *P. gingivalis*, but recent research indicates that *P. gulae* is also found in humans with periodontal disease,^20^ and is able to adhere to and invade human gingival epithelial cells.^21^ Importantly, *P. gingivalis* and *P. gulae* are the only bacterial species known to produce gingipains, which include the arginine‐gingipains RgpA/B in addition to Kgp.^22^ Oral biofluid sampling in dogs enabled us to analyze COR388 Kgp target engagement and pharmacologic effects on *P. gulae* bacterial load at multiple time points and doses. These data were used to understand the pharmacology of COR388 in naturally infected tissues and biofluids of the oral cavity. In oral tissues, systemic COR388 treatment reduced Kgp activity and *P. gulae* levels over a 28‐day period in a dose‐dependent manner. Based on these data, the lowest effective dose of COR388 was chosen for administration for 90 days, and this dose demonstrated efficacy in reducing periodontal disease pathology.

In addition, we demonstrate that *P. gulae* DNA and Kgp antigens are present in the aged dog brain, with similar histopathology to what we identified for *P. gingivalis* and gingipains in the human AD brain.^1^ Thus, the preclinical work with COR388 reported here allowed us to pharmacologically evaluate the drug's ability to inhibit Kgp, reduce *P. gulae* bacterial burden and disease pathology in a naturally occurring infection in dogs, providing proof‐of‐concept for targeting Kgp and *P. gingivalis* in human studies.

## 2 METHODS

### 2.1 Dog pharmacology model

Aged Beagle dogs of both sexes ranging from 8 to 15 years of age, in generally good health and located in the Vivocore Inc dog colony, were utilized in this study. All procedures were reviewed and approved by Vivocore's Institutional Animal Care and Use Committee (IACUC) and were performed in accordance with the principles of the Animal for Research Act of Ontario and the guidelines of Canadian Council on Animal Care (CCAC). Structure‐based design was used to develop a library of gingipain inhibitors, which were tested on purified Kgp and RgpB to assess potency and determine inhibition constants. The detailed chemical synthesis and structure of compounds in the relevant series of lysine‐gingipain inhibitors including COR388 can be found in PCT/2016/0061197. Detailed characterization of COR388 enzyme target binding, potency, and selectivity are previously described.^1^ Test article administered, COR388 HCl, was a solid powdered material. Dogs were dosed with neat compound hand‐filled in gelatin capsules (size 1, Torpac) orally at the listed doses (free‐base, corrected for salt form). All dogs utilized in studies were positive on previous BANA enzyme (BANAMet LLC, Ann Arbor, MI) testing of oral plaque samples (assay described below). In the first study, four dogs were dosed 10 mg kg^−1^ q.d. with endpoints assessed after dosing and compared to those taken pre‐dose in individual dogs. The second dose‐response study was conducted with a randomized, blinded, matched‐group design with 12 dogs. Following baseline sample collections prior to dosing, animals were allocated to four dosing groups, n = 3 dogs per group as follows: vehicle (empty capsule), 0.15, 0.5, and 1.5 mg kg^−1^ b.i.d (Q12h). Dosing continued for 28 days (0.15 and 1.5 mg kg^−1^) or 90 days (vehicle and 0.5 mg kg^−1^). Groups were balanced for age, sex, weight, and baseline BANA test results to the extent possible. Dogs were group housed according to dosing group in compliance with the recommendations of the CACC. Dogs were fed a standard commercial dry diet daily in the evenings with water provided ad libitum but not within 30 minutes of the start of anesthetic procedures*.*

Gingival crevicular fluid (GCF), subgingival plaque (SGP), buccal cells, saliva, and plasma samples were collected and BANA enzyme testing in the oral cavity was performed throughout the studies. Procedures for oral biomarker sample collection are described below and were performed under general anesthesia induced with 8 mg kg^−1^ of propofol (intravenous) to effect. Following intubation, anesthesia was maintained with an isoflurane‐oxygen mixture. Procedures on study Day 1 were performed under sedation with medetomidine (reversed with atipamezole), as full anesthesia was not required for the collections performed on this day.

For evaluation of pharmacokinetics parameters, approximately 2 mL of blood was collected at the indicated time points following test article administration into K_2_EDTA tubes and plasma was isolated for bioanalytical determination of plasma concentrations of COR388. A single intravenous dose of COR388 was administered in PBS at a dose of 1 mg kg^−1^. Quantitation of COR388 in plasma samples following protein precipitation with mianserin added as an internal standard, was performed from the time points shown using a qualified LC‐MS/MS method (AB Sciex 4000 QTrap). Plasma concentration versus time profiles were analyzed to determine pharmacokinetic parameters using noncompartmental methods (Phoenix^®^ WinNonlin^®^ 6.3, Pharsight Certara).

### 2.2 BANA enzyme test

The test provided a qualitative assessment of arginine‐gingipain protease activity derived from oral bacteria by assessing proteolytic cleavage of N‐benzoyl‐dL‐arginine‐2‐napthylamide substrate. Subgingival plaque was collected using a dental scaler from the base of two teeth above the gum line. Plaque samples were applied to a BANA test strip and analyzed with the manufacturer's instructions (BANAMet LLC).

### 2.3 Bacterial strains used and growth conditions

*P. gingivalis* W83, *P. gingivalis* ATCC 33 277 and *P. gulae* ATCC 51 700 (ATCC, VA) bacteria were grown in modified tryptic soy broth media (TSB media supplemented with 5 mg mL^−1^ yeast extract, 5 μg mL^−1^ hemin, 1 μg mL^−1^ menadione, 0.5 mg mL^−1^ L‐cysteine) to late mid‐log phase, collected by centrifugation for 20 minutes at 4000 *g*, washed in PBS, then resuspended in PBS. Fifty microliters containing approximately 10^6^ CFUs bacteria was used for each Kgp activity assay, for potency determination from intact bacteria. To prepare lysates for analysis, bacteria were washed in PBS and lysed in B‐Per lysis buffer (Thermo Fisher Scientific) on ice for 20 minutes, centrifuged for 30 minutes at 16 000*g*, 4°C, and supernatant lysates collected. Protein concentration was measured using Pierce BCA protein assay kit (Thermo Fisher). When lysates were used for Kgp activity assays, 500 ng of bacterial lysate was used for the assay.

### 2.4 Kgp enzyme inhibition assay

Fifty microliters of each sample (intact bacteria or 500 ng bacterial lysate) was added to a transparent, black 96 well plate and COR388 stock solution in PBS was added to samples for final concentrations of 300, 100, 30, 10, 3, 1, 0.3, and 0 nmol L^−1^. Assay plates were covered in film with shaking for 30 seconds before incubation at 37°C for 30 minutes. Fifty microliters of 2X protease substrate Z‐His‐Glu‐Lys‐MCA (Millipore Sigma), to yield a 10 μmol L^−1^ final concentration in KgP assay buffer (100 mmol L^−1^ Tris, 75 mmol L^−1^ NaCl, 2.5 mmol L^−1^ CaCl_2_, 10 mmol L^−1^ Cys‐HCl), was added to each sample. Enzyme activity was then immediately measured in a Synergy 2 microplate reader (Biotek) at 380/460 nm excitation/emission wavelength, read every 1.5 minutes for 30 minutes with incubation and shaking at 37°C. Enzyme activity data were analyzed using Gen5 software (Biotek).

### 2.5 Kgp enzyme inhibition assay in bacterial cultures using an activity‐based probe

5 × 10^6^ CFU in PBS were incubated with COR388 at the concentrations ranging from 0.3 to 1000 nmol L^−1^ 37°C for 30 minutes, followed by the addition of 1 μmol L^−1^ COR553 Cy5‐labeled activity probe to bind remaining active sites and incubated at 37°C for another 30 minutes. The COR553 activity probe was prepared by the copper‐catalyzed azide‐alkyne cycloaddition reaction between an azide derivative of the irreversible Kgp inhibitor COR553 and an alkyne amide derivative of the Cy5 fluorophore. The probe forms an irreversible covalent bond with a catalytic cysteine residue in the active site of Kgp by displacement of a phenol leaving group, and its use has been previously described in detail.^1^ Sample loading dye was added, samples heated at 95°C for 5 minutes, and analyzed on XT Criterion gels (Biorad). Cy5 probe labeled protein was detected using a Chemidoc imaging system (Biorad) and quantified by Imagelab software (Biorad). A rabbit polyclonal anti‐Kgp antibody, CAB102, described previously,^1^ was used to detect Kgp by Western Blot analysis to ensure equal loading of bacteria and presence of the enzyme. This sample loading was confirmed in each experiment with one example included.

### 2.6 Oral biofluid sample collection

Saliva: Saliva was collected using a SalivaBio collection swab (Salimetrics LLC) on the mouth interior. The swab was inserted into the saliva collection vial, centrifuged at 1500 *g* for 15 minutes at room temperature. The swab was then removed from the vial and the liquid sample was stored as aliquots of 50‐100 μL at −80°C prior to analysis.

Subgingival plaque: SGP was collected from eight different gingival pockets per dog using Absorbent Points (Coarse, Premier^®^ Dental Products Company, Plymouth Meeting) at times following dosing ±30 minutes, as indicated. Four gingival pockets were used per timepoint. Paper points were inserted into gingival pockets (held by tweezers) until resistance was felt and held for 30 seconds. Four paper points were individually washed with 100 μL PBS‐0.05% Tween for 30 seconds and eluate placed into a 1.5 mL microcentrifuge tube. Eluate samples were stored at −80°C prior to analysis.

Gingival Crevicular Fluid: GCF was collected from the gingival pockets at times following dosing ±30 minutes, as indicated. Eight sampling sites were identified per dog and four sites were used per timepoint. Sampling sites were isolated with a cotton roll and air‐dried. Gingival fluid collection strips (Periopaper™, Oraflow) were gently held with tweezers and placed into the pocket until resistance was felt. Strips were held in place for 30 seconds and then processed as described above for SGP isolation.

Buccal cell: Four buccal swabs were performed on each dog by swabbing the inside of the cheek with an oral brush biopsy sampling instrument (OralCDx, Suffren) before dosing as well as day 14 and 28 and for some dogs on day 48 at times following dosing ±30 minutes, as indicated. Two brushes were washed one at a time, in a 15 mL falcon tube containing 1.5 mL of DMEM, solution transferred to a 1.5 mL microfuge tube, and centrifuged at 1500 *g* for 10 minutes at room temperature. Most of the supernatant was aspirated and the pellet was snap frozen. Procedures were repeated for the remaining two oral brushes.

### 2.7 qPCR detection of *P. gulae* in saliva, buccal cells, and GCF

Biofluids were evaluated for *P. gulae* load using a qPCR assay that was developed to specifically detect *P. gulae* 16S gene with the following primers and probe: Forward primer CGAGGGGCAGCATGAACTTA, Reverse primer TTGCCCGATCATGCAACCAA, and probe GCGTAACGCGTATGCAACTTGCCTTAC. Primers were designed from regions of *P. gulae* 16S that have sequence mismatches with *P. gingivalis* sequence to ensure a specific detection of *P. gulae*. Saliva was evaluated with either 2 μL neat saliva or purified DNA from 2 μL saliva. Buccal cells were evaluated with either 2 μL neat buccal cell slurry or purified DNA from 2 μL buccal cell slurry. SGP was evaluated with 2 μL from the elution in B‐Per. GCF was evaluated with either purified DNA from 50 μL of B‐Per eluted GCF or neat 2 μL elution.

### 2.8 Kgp enzyme activity analysis in GCF and SGP using an activity‐based probe

Twenty microliters of neat saliva, SGP and GCF eluates and 50 μL lysed buccal cell slurry were incubated with the COR553 activity probe as described above, at a final concentration of 1 μmol L^−1^. Probe binding analysis was performed as described above. BCA protein detection assay results were used to equally load the gels. Active Kgp was extrapolated from the purified Kgp (purified Kgp was a kind gift of Barbara Potempa, University of Louisville) standard curve that was run on each gel. To normalize samples run on separate gels, the extrapolated Kgp value was divided by the ng of total protein loaded per lane since the value varied between gels.

### 2.9 Rgp enzyme activity assay in SGP

For the detection of arginine‐gingipain (Rgp) activity, cysteine‐HCl was freshly added to RgpB buffer (100 mmol L^−1^ Tris, 200 mmol L^−1^ gly‐gly, 5 mmol L^−1^ CaCl_2_) to a final concentration of 10 mmol L^−1^ to produce a RgpB working enzyme assay buffer. Purified RgpB (purified RgpB was a kind gift from Barbara Potempa, University of Louisville) was thawed on ice and was added to RgpB working buffer with 2× concentration to prepare standards of final concentration from 10 nmol L^−1^ to 0.0156 nmol L^−1^. Substrate L‐BapNa (Sigma) from stock of 200 mmol L^−1^ in DMSO was added to a separate tube of RgpB working buffer to make concentration of 2 mmol L^−1^ to make substrate solution. Ten microliters of dog plaque samples prepared in B‐per solution (Thermofisher) was diluted with 40 μL of RgpB working solution to be used for the assay. 50 μL of 2× enzyme standards and 50 μL of sample solutions were added to a pre‐cooled 96 well, clear bottom, black plate (Greiner Bio‐One) on ice and appropriate blanks were added. After 50 μL of substrate solution was added, the plate was immediately read at 405 nm for 1 hour with interval of every 1.5 minutes read at 37°C and continuous shaking in Biotek Synergy 2 microplate reader. COR286, an RgpB inhibitor previously described,^1^ was used to assess background activity in baseline samples and any enzyme activity seen not due to RgpB activity was subtracted from the assay. Detailed chemical synthesis and structure of compounds in the relevant series of arginine gingipain inhibitors including COR286 can be found in International Patent Application PCT/US2015/054050 and PCT/US2016/061197.

### 2.10 Dental assessments

Assessments were performed 2 hours following the evening dose time 7 days prior to the start of dosing (predose) and also included on days 27, 28, 29, 91, and 92. These included an examination of pocket depth (listed in mm). Dental assessments were performed after all biomarker samples were collected so as not to disturb biofluids with this assessment.

### 2.11 Immunohistochemistry analysis of gingipains in dog brain tissue

Immunohistochemistry (IHC) staining was performed on postmortem brain samples from four dogs not utilized in the described pharmacology studies. Kgp was detected using polyclonal antibody generated to a peptide from lysine‐gingipain CAB102 as previously described.^1^ Antibody specificity was confirmed through IHC staining of bacteria‐positive gingival tissues and pre‐absorption of the antibody to Kgp eliminated the brain tissue staining in dog brain tissues.^1^ Sections were deparaffinized in xylenes and alcohol series and submitted to heat mediated antigen retrieval in citric acid at pH 6.0 (Vector Laboratories, Burlingame CA). Sections were blocked in 5% normal horse serum with 0.3% triton‐x‐100 in PBS and incubated overnight at 4°C in primary antibody 1:2000 in 2.5% NHS and 0.15% triton‐x‐100. After washing three times in PBS, Impress HRP polymer detection kit and Impact DAB EqV substrate were used to visualize the primary antibody binding. Images were taken on an Olympus motorized microscope BX61 equipped with a color CCD camera (Olympus, DP27) and processed for brightness and contrast correction, cropping and addition of scale bars with CellSens 1.14 Dimension software.

### 2.12 PCR detection and sequence analysis of bacteria in aged dog brain tissue

Frozen brain tissue samples from the same postmortem dog samples used for IHC analysis of Kgp protein were also analyzed for the presence of *P. gulae* bacteria by qPCR followed by sequence analysis of the PCR product. qPCR copy number detected is shown normalized per brain cell number listed knowing the quantity of DNA utilized in the PCR reaction and average DNA per cell. Hippocampus was dissected from the frozen brain, the weight measured and then homogenized in 1 mL of ice cold RIPA buffer (VWR Life Science RIPA Lysis Buffer) with the Qiagen TissueLyser II at 30 Hz for 2 minutes. Protein lysate (200 μL) was immediately transferred into 1 mL cold Trizol solution (TRI reagent, Millipore). After removal of the aqueous phase (standard TRI Reagent RNA extraction protocol, Millipore), the DNA in the phenol phase was precipitated with 100% ethanol, centrifuged at 13 500 *g*, washed again with 75% ethanol, and air dried for 10 minutes. To ensure protein removal, the pellet was solubilized in ATL buffer (Qiagen) and digested over night with Proteinase K. DNA purification was performed according to the instructions of the Qiagen DNeasy Blood &amp; Tissue Kit. DNA was eluted from the Qiagen column in 60 mL of TE buffer. DNA (40 ng) was used for qPCR by the method described above for biomarker qPCR evaluations.

### 2.13 Data and statistical analysis

IC_50_ values were extrapolated using a non‐linear fit for the data plotted with log(conc) vs normalized response and the 95% confidence interval (CI) is shown for each data point (average of N = 3 replicates) using GraphPad Prism 7. The normalized response for the IC_50_ determination was performed with respect to no inhibitor control that was set to 100% Kgp activity. The curve fit to the data was evaluated by the *R* ^2^ value, which was ≥.9 for all the IC_50_ curves. All PK graphs (statistical significance in GraphPad Prism 7) and parameters (statistical significance in WinNonlin) are shown as mean with standard deviation (SD). All target engagement data in biomarker samples is presented as the mean with standard error of the mean (SEM) determined using the Unpaired *t*‐test in GraphPad Prism 7. All values presented as % of baseline were calculated by setting the baseline to 100% response and statistical significance is shown compared to baseline. Statistical significance where specific groups on a graph were compared is shown by bracketed horizontal lines. The asterisk convention to denote *P* value was followed: *P < .05; **P < .01; ***P < .001; ****P < .0001.

## 3 RESULTS

### 3.1 Lysine‐gingipains from *P. gingivalis* and *P. gulae* have similar proteolytic activity

Kgp from *P. gulae* and *P. gingivalis* have similar amino acid sequences and specific activity, suggesting similar functionality on substrates.^22^ Using in vitro substrate cleavage assays, we characterized the specific activity of Kgp from two species of *P. gingivalis* as well as *P. gulae*. Proteolytic activity using the Z‐His‐Glu‐Lys‐MCA peptide substrate was assayed from bacterial cultures to assess activity of bacterial surface‐associated Kgp as well as bacterial lysates in a buffer which preserves protease activity. Values for maximal velocity (V_max_), Kgp concentration, and specific activity (rate of proteolysis) were similar between two strains of *P. gingivalis* and *P. gulae* (Table 1). These data suggest that these Kgp enzymes have similar structure and specific activity and indicate COR388 may have potency across these bacterial species and strains.

**Table 1:** *Porphyromonas gingivalis* and *Porphyromonas gulae* Kgp proteases share similar gingipain catalytic activity

Bacteria		Substrate assay *V*_max_ (min^−1^) (10^7^) (95% CI)		Substrate assay [Kgp] (nmol L^−1^) (95% CI)		Specific activity (*V*_max‐_Kgp^−1^ nmol L^−1^) (10^7^)	
		Intact Pg (5 × 10^6^ cfu)	Pg lysate (500 ng)	Intact Pg (5 × 10^6^ cfu)	Pg lysate (500 ng)	Intact Pg (5 × 10^6^ cfu)	Pg lysate (500 ng)
*P. gingivalis*	ATCC 33277	1.67 (1.49‐1.88)	2.08 (1.92‐2.24)	1.61 (1.31‐1.90)	0.80 (0.74‐0.85)	1.04	2.6
	W83	2.56 (2.16‐2.95)	1.45 (1.22‐1.69)	1.43 (1.07‐1.78)	0.72 (0.58‐0.85)	1.79	2.02
*P. gulae*	ATCC 51700	2.08 (1.92‐2.24)	1.39 (1.29‐1.48)	2.02 (1.61‐2.43)	0.88 (0.49‐1.27)	1.03	1.58

Note

Protease activity in intact bacterial culture for the three strains listed and separately from lysates prepared from these bacteria was measured as described in Methods. The specific activity was determined by dividing the protease activity *V*_max_ with the concentration of Kgp identified in the assays.

### 3.2 COR388 potently inhibits lysine‐gingipain from both *P. gingivalis* and *P. gulae*

**Figure 1:**
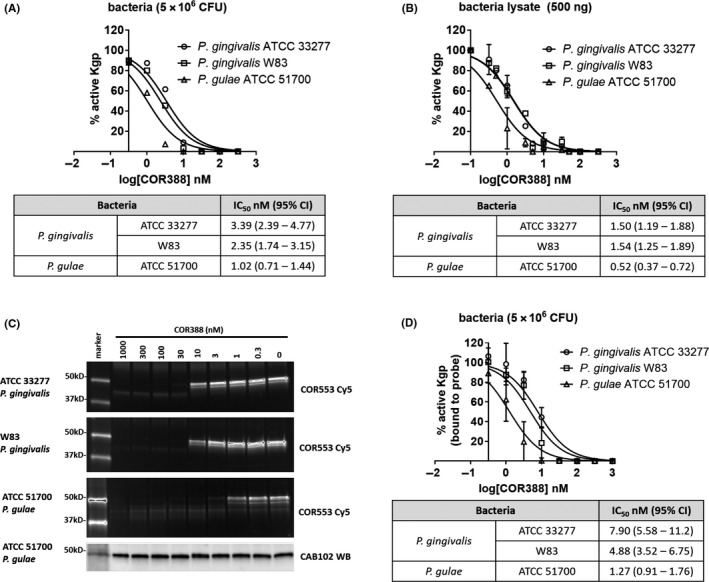
COR388 has similar potency on the Kgp protease from both *Porphyromonas gingivalis* and *Porphyromonas gulae*. (A) Bacterial surface‐associated Kgp activity from growing cultures was assayed with a substrate cleavage assay (substrate Z‐His‐Glu‐Lys‐MCA) on intact bacteria; (B) Bacterial lysate prepared from growing cultures was assayed for Kgp activity with the same substrate cleavage assay; (C and D) Bacteria‐associated Kgp activity was assayed from growing cultures using an active site‐binding activity‐based probe (COR553‐Cy5). Intact buffer‐washed bacteria were incubated with serial dilutions of COR388 for 30 minutes and remaining Kgp was detected by adding activity‐based probe COR553 for 1 hour to bind remaining unbound Kgp. Proteins were separated biochemically and visualized by imaging the Cy5 fluorescence, or total Kgp protein present verified by immunoblotting with CAB102 anti‐Kgp antibody, with one example immunoblot shown in C. The percent of active Kgp remaining (probe bound) was calculated relative to the maximal probe binding in the absence of COR388. Quantitation of C is shown in D. IC_50_ values in all three assays were determined through serial dilutions of COR388 and assessing remaining protease activity. Inhibition constants are calculated as an IC_50_ value and the 95% confidence intervals listed with each study performed in triplicate

The inhibition of Kgp protease activity from *P. gingivalis* by COR388 and the kinetics of binding were previously reported.^1^ In order to confirm the potency of COR388 on Kgp expressed by *P. gulae*, three in vitro assays were conducted. Potency was assayed on intact bacterial surface‐associated Kgp (Figure 1A) and on a bacterial lysate (Figure 1B). Potency of inhibition was single digit nmol L^−1^ or lower in both assays and similar between strains. Inhibition of Kgp was also assayed with an activity‐based probe binding assay. COR553 is an irreversible active‐site Kgp probe labeled with Cy5 for detection, previously described.^1^ Bacterial cultures were treated with COR388 for 30 minutes, followed by exposure to the COR553 probe in which all Kgp active sites remaining unbound by COR388 are bound by the probe. Samples were biochemically separated and active Kgp detected with Cy5 fluorescence and total Kgp protein detected by immunoblotting with CAB102 Kgp‐specific antibody (Figure 1C and D). IC_50_ values were similar between strains and species and remained single digit nmol L^−1^ in potency. These data confirm the potency of COR388 for the Kgp virulence factor of *P. gulae*.

### 3.3 COR388 is orally bioavailable in dogs with moderate metabolic stability

**Figure 2:**
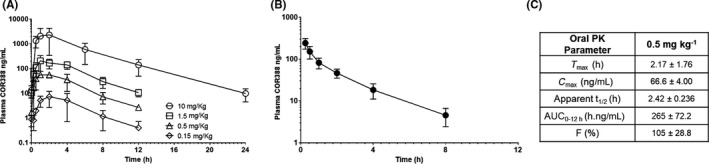
COR388 pharmacokinetic (PK) profile and parameters with oral and intravenous administration. (A) Plasma PK profile of listed single oral doses (10, 1.5, 0.5, and 0.15 mg kg^−1^) administered in gelatin capsules. (B) Plasma PK profile of single intravenous administration (1 mg kg^−1^) administered in phosphate buffered saline. (C) PK parameters following a single oral dose were calculated using WinNonlin and are shown where *T*_max_ is the time to achieve maximal plasma concentration; *C*_max_ is the maximal concentration achieved; Apparent *t*_1/2_ is the time to reduce maximal concentration by half; AUC_0‐12h_ is the area under the curve for 12 hours after administration; and *F* is the bioavailability assessing the fraction of the drug reaching systemic circulation

Plasma concentrations of COR388 were measured after oral and intravenous administration using LC‐MS/MS methods. The pharmacokinetic profile of oral doses of COR388 administered in gelatin capsules is depicted in Figure 2A and after a single intravenous dose of 1 mg kg^−1^ COR388 in Figure 2B. Following intravenous dosing, a moderate systemic clearance (CL_S_ = 2.1 ± 0.6 L h^−1^ kg^−1^) and large volume distribution (V_ss_ = 5.2 ± 1.3 L kg^−1^) for COR388 was observed, resulting in an elimination half‐life of 3.5 ± 0.6 hours. Excellent bioavailability (%F = 100% at doses of 0.5 mg kg^−1^ and higher) and exposure that was proportional to dose was also observed for the 0.15 to 10 mg kg^−1^ oral doses. Mean pharmacokinetic parameters for the 0.5 mg kg^−1^ dose are summarized in Figure 2C. At this dose administered b.i.d. for 6 days, a C_max_ value of 66.6 ng mL^−1^ was observed at 2.17 hours after dosing, with an AUC_0‐12 h_ of 265 h ng mL^−1^. The mean trough plasma concentration (*C*_min_) value 12 hours after oral dosing of 0.5 mg kg^−1^ was 2.67 ng mL^−1^.

### 3.4 Oral administration of COR388 for 28 days results in target engagement and sustained reduction in *P. gulae* bacterial load

Periodontal disease mediated by pathogenic bacteria is naturally occurring in dogs and has a similar disease course and microbiology as that found in humans.^23^, ^24^, ^25^ The natural infection of *P. gulae* in dogs with readily accessible biofluids for bacterial analysis presents an opportunity to analyze COR388 pharmacology and in vivo potency in a naturally occurring infection. A colony of aged Beagle dogs in which periodontal disease and *P. gulae* infection was naturally occurring was utilized to assess the efficacy of COR388 in engaging the target and modulating bacterial load. Target engagement of COR388 with the Kgp active site in vivo and its effect on *P. gulae* copy number in oral biofluids was assessed in aged dogs ranging from 10.3 to 15.4 years of age, following oral COR388 administration at a dose of 10 mg kg^−1^ once a day for 28 days. Biofluids assessed included saliva, buccal cells, subgingival plaque (SGP), and gingival crevicular fluid (GCF). Changes in Kgp activity, arginine‐gingipain (RgpB) activity, and *P. gulae* bacterial load were measured from baseline pre‐dose levels in individual dogs. All dogs were confirmed positive at baseline for periodontal disease‐associated bacteria with a commercially available diagnostic for arginine‐gingipain activity (BANA test), which confirms the presence of a *Porphyromonas* infection.

**Figure 3:**
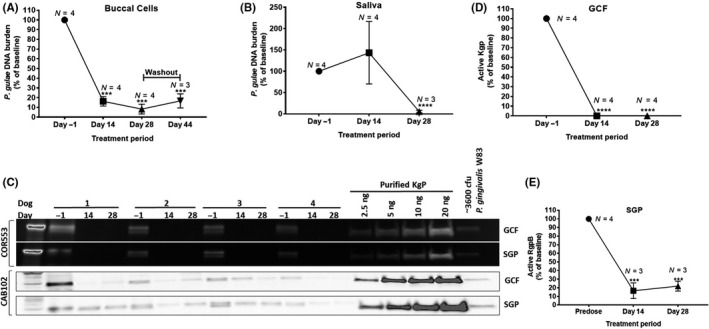
COR388 oral administration at a dose of 10 mg kg^−1^ results in sustained decrease in gingipain activity and bacterial burden. (A) Dogs were dosed from day 1 to day 28; sample was taken 16 days following cessation of dosing on day 44. (B) *P. gulae c*opy number from 2 mL saliva normalized to predose. (C) Kgp activity was assayed in GCF and SGP using activity‐based COR553 probe as described in Methods. Kgp protein was assayed by a Western blot using the polyclonal CAB102 antibody. Purified Kgp and *P. gingivalis* W83 lysates were run as positive controls. (D) Levels of Kgp activity normalized to individual predose values were quantified from C. (E) RgpB substrate cleavage assay in SGP samples, with values normalized to predose levels. Significance was determined using a *t*‐test and mean ± SEM values are shown

Buccal cells, known to harbor intracellular *Porphyromonas* infection, were assayed for *P. gulae* bacterial load using qPCR. Buccal cells were collected at baseline, day 14, and day 28 of dosing. Dosing was stopped on day 29, followed by a period with no dose administration (to day 44). A mean decrease of > 80% in baseline *P. gulae* DNA was observed in buccal cells after 14 days of dosing, dropping further on day 28 (Figure 3A). A small recovery of bacterial load was observed on day 44 (Figure 3A) indicating a durable response in reducing the bacteria, but also a slow recovery due to either reinfection or proliferation of remaining bacteria. These data support the need for chronic dosing to achieve sustained reduction of *P. gulae* and for the potential of therapeutic efficacy. Saliva samples were also assayed for *P. gulae* load using qPCR after 14 and 28 days of dosing. While the level of the bacterial load in saliva was more variable than that observed in buccal cells, a similar reduction from baseline levels was observed after 28 days of COR388 administration (Figure 3B).

GCF collected on and eluted from paper strips was also assayed for *P. gulae* DNA by qPCR and Kgp activity using the activity‐based active site probe COR553. Kgp activity was robustly detected with this method, consistent with the presence of the bacteria. COR553 probe‐bound Kgp was present at baseline predose on day 1 and reduced in samples taken 2 hours after dosing on day 14 and day 28 (Figure 3C). These data indicate the presence of active Kgp available for probe binding in all dogs on day 1 and indicate that no active sites were available for probe binding on days 14 and 28. Quantitation of the Kgp protein was performed using reference standards of purified Kgp also bound to the active site probe. The level of reduction in active Kgp from baseline is quantified in Figure 3D. Reduced levels of the total Kgp protein, detected with a polyclonal anti‐Kgp CAB102 antibody, were seen in these same lysates following COR388 administration on days 14 and particularly on day 28, suggesting that the Kgp protein in total was reduced. There is no loading standard for GCF and therefore relative comparisons to untreated samples were performed. This reduction is consistent with the results of reduced *P. gulae* bacterial burden observed in buccal cells and saliva and is consistent with the inhibition of Kgp and subsequent clearance of the *P. gulae* infection following administration of COR388.

SGP was also collected and assayed for *P. gulae* DNA by qPCR and Kgp activity using the COR553 probe. Kgp activity is significantly reduced with COR388 administration similar to the data in GCF (Figure 3C). In addition, RgpB protease activity was assayed using a substrate cleavage assay in SGP as described in Methods. Active RgpB was detected at baseline on day 1 with a reduction following COR388 administration (Figure 3E). This is consistent with the reduction in *P. gulae* levels also observed, as levels of all bacterial‐produced proteins would likewise be expected to fall concurrent with a reduction in bacteria.

Together these data on multiple readouts of gingipain activity and *P. gulae* load, from independent oral biofluids harboring the bacteria, are consistent with COR388 target engagement on Kgp and subsequent reduction in bacterial burden.

### 3.5 Oral administration of COR388 results in dose‐dependent Kgp target engagement

Kgp active site inhibition as well as a robust anti‐bacterial response was observed in the first study. A second study in an independent cohort of aged dogs was performed to assess the dose‐response relationship of COR388 including a vehicle control. In this second study, 12 aged dogs 8.6‐14.6 years of age, identified as BANA‐test positive, were randomized into four study groups balanced for age, sex, weight and baseline BANA results. Dogs were dosed with COR388 in capsules including a vehicle group (empty capsule) with 0.15, 0.5, and 1.5 mg kg^−1^ b.i.d. dose groups. Having demonstrated similar target engagement and reduction of *P. gulae* DNA in multiple biofluids in the first study, biomarker analysis in this second study was focused on a subset of biomarkers and biofluids to be able to analyze more time points as well as periodontal disease endpoints. In this study GCF was analyzed for detection of Kgp activity and buccal cells were analyzed for *P. gulae* DNA quantitation. DNA purification and normalization to GAPDH DNA was added to the buccal cell analysis for normalization and quantitation of bacterial copy number. GCF and buccal samples were collected as described in Methods and biomarkers evaluated in all dogs prior to dosing, multiple times after the first dose, and at single time points on days 14 and 28 of the study. Collection 12 hours after the first dose was included to assess biomarker activity at the plasma pharmacokinetic trough concentration (*C*_min_).

**Figure 4:**
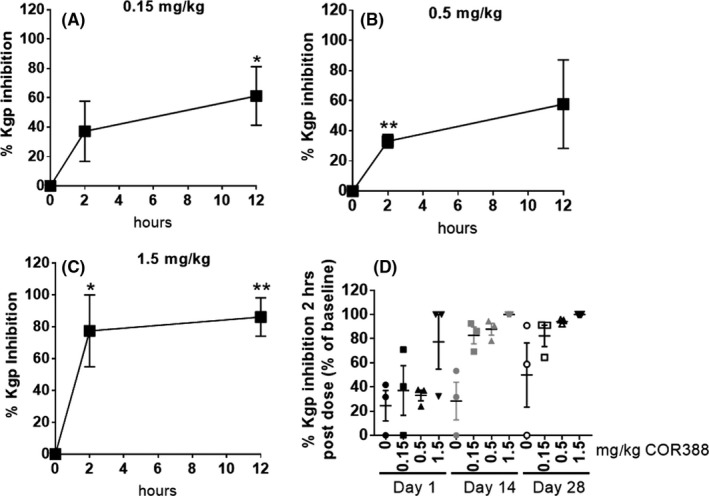
COR388 inhibits Kgp in a dose and time dependent manner. (A‐C) Target engagement at 0 (predose), 2 and 12 hours after the first dose on day 1 was measured using COR553 probe binding and quantitated by densitometry to measure inhibition in all dosing groups. Fluorescent probe images from Figure S1 were quantitated and ng of Kgp was determined by the purified Kgp standard curve. Percent inhibition from baseline (normalized to 0% inhibition) is shown, plotting mean ± SEM. (D) The time course of this target engagement in GCF (percent inhibition from baseline) for all doses, is shown on days 1, 14, and 28

Kgp activity in GCF was again assessed using the COR553 probe. Early time points allow assessment of target engagement in which the bacteria and Kgp protein are not yet cleared. As expected, strong probe binding was detected in baseline samples. Kgp activity was inhibited by COR388 in a dose‐dependent manner (Figure 4; Figure S1) indicating active site target engagement by COR388. Kgp inhibition increased between 2 and 12 hours after the first dose and was maximal at the highest dose administered (Figure 4A‐C). Furthermore, the level of Kgp inhibition increased with repeated dosing for 28 days resulting in a greater level of inhibition over time (Figure 4D), likely also reflecting a reduction in *P. gulae* and Kgp levels. These studies demonstrated a dose‐dependent decrease in Kgp activity 2 hours post dose (near the plasma T_max_ exposure) with negligible recovery of activity indicating continued target engagement and inhibition for 12 hours following the dose (at C_min_) (Figure 4A‐C and Figure S1B‐E). While variable between dogs, inhibition at all doses generally increased with increasing time of exposure on day 1 (Figure S1F‐H). It was noted that one dog receiving a dose of 0.15 mg kg^−1^ had minimal Kgp inhibition on all days and this dog had the lowest exposure of all dogs with a plasma C_max_ of only 2.73 ng mL^−1^ compared with the mean C_max_ of 9.74g mL^−1^ in that dose group, consistent with a dose‐response relationship of Kgp inhibition. While some natural variability in Kgp activity can be seen over time, inhibition of Kgp activity in GCF was not detected in the vehicle‐treated dogs (Figure S1A and E). These data support dose‐dependent inhibition of Kgp in oral biofluids following administration of COR388.

### 3.6 Extended dosing of COR388 for 90 days is efficacious in reducing pocket depth in dogs

**Figure 5:**
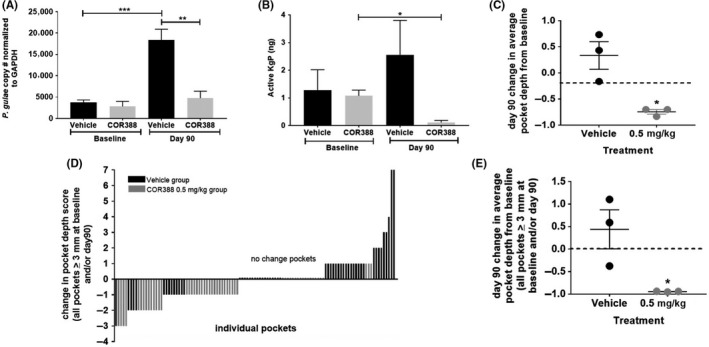
COR388 administered 0.5 mg kg^−1^ b.i.d. resulted in pharmacologic efficacy in periodontal disease. (A) *P. gulae* copy number using 16S primers in DNA isolated from buccal cells at baseline on day 1 and day 90 is shown, normalized to cellular GAPDH DNA levels. (B) Normalized Kgp activity (ng) in GCF at baseline on day 1 and on day 90 is shown. The method for normalization is described in the methods section. (C) Change in average pocket depth from baseline on day 90 is shown where each dot represents the numerical change (mm) from baseline measured for each dog. Negative change in pocket depth indicates improvement. (D) Waterfall plot for change in individual pocket depths for all pockets in vehicle and COR388 groups that were either a pocket depth of 3 mm or greater at baseline or day 90. Equal number of pockets were assayed for COR388 and vehicle groups. (E) All pockets in vehicle and COR388 groups that were either a pocket depth of 3 mm or greater at baseline or day 90 were averaged for each dog and that average per dog was plotted and grouped in either a vehicle or COR388 group for graphing. This graph is a subset analysis of graph C and the data points used are individually plotted in graph D. Statistical significance shown based on standard *t*‐test using Graphpad PRISM, with plots showing mean ± SEM

Having established target engagement and inhibition in a dose‐dependent manner, one dose was chosen to continue for 90 days to determine if extended dosing would result in a prolonged reduction of *P. gulae* bacterial burden and efficacy in reducing periodontal disease pathology. Pocket depth, a measure of gingival inflammation and clinical attachment loss (CAL) associated with periodontal disease, was assessed in the dogs prior to the start of dosing and measured after 90 days of administration. Near‐complete Kgp inhibition in all three dogs was detected over 28 days using the 0.5 mg kg^−1^ dose and therefore, this dose was extended along with the vehicle control group for this 90‐day study. COR388 plasma exposure for this dose was similar to that seen with a maximally efficacious dose in a mouse model of *P. gingivalis* infection.^1^ Monitoring of Kgp activity and *P. gulae* in GCF samples was continued in these dose groups. COR388 administration resulted in statistically significant reduction in *P. gulae* bacterial load over time as well as Kgp activity when compared to the vehicle control animals (Figure 5A and B). The data suggest that continued COR388 administration kept bacterial burden reduced in contrast to the rise in *P. guale* load in the vehicle dosed group.

Gingival pocket depths were measured from 10 teeth from three pockets (Rostral, Lateral, and Caudal) on each tooth. Pocket depths were reduced with COR388 administration, indicative of reduced periodontal disease and associated inflammation. In contrast, the vehicle dosed group progressed with increases in pocket depth. There was a reduction in mean average pocket depth with COR388 administration when changes in all individual pocket depths were measured (*P* = .0156) (Figure 5C). Pocket depths in this model ≥3 mm are consistent with periodontal disease pathology. A waterfall plot of the change in pocket depth size from day 1 to day 90, for all pockets ≥3 mm at baseline or on day 90, illustrated that the pockets that decreased were primarily in animals administered COR388 (Figure 5D). A significant reduction in mean average pocket depth ≥3 mm at baseline or end of study per dog with COR388 dosing was seen when this subset of most‐effected pockets was analyzed (*P* = .0332) (Figure 5E). By contrast, pocket depth tended to increase in dogs receiving the vehicle control.

### 3.7 Detection of Kgp antigens and identification of *P. gulae 16S rRNA* gene in aged dog brains

**Figure 6:**
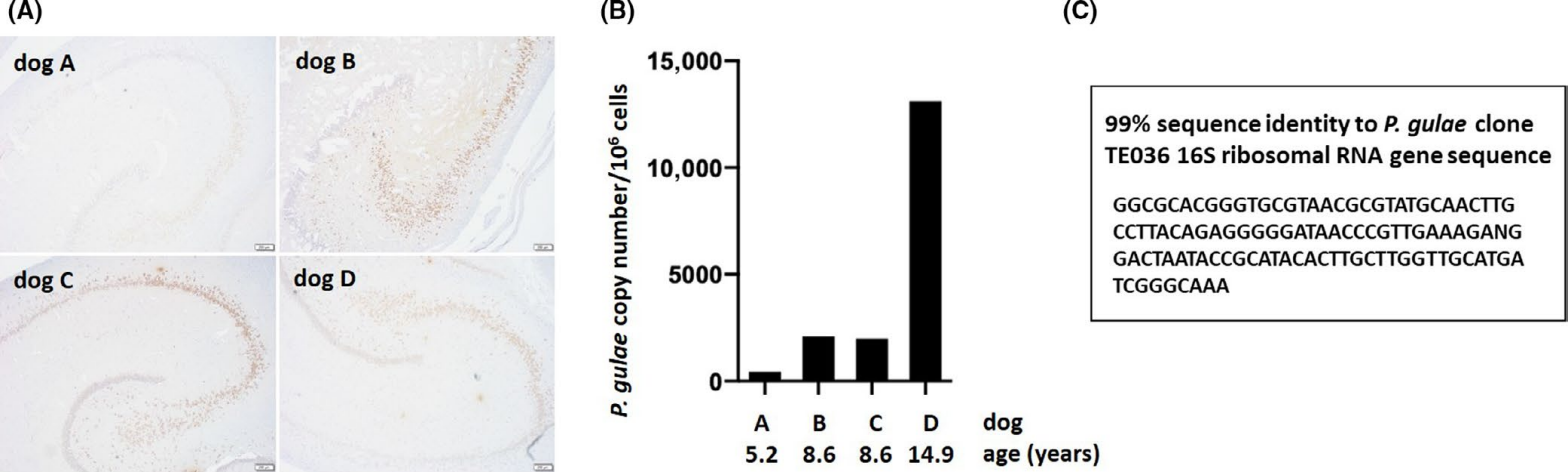
Detection of Kgp antigens and *P. gulae* DNA in aged dog hippocampal samples. (A) Kgp IHC with polyclonal antibody CAB102 on dentate gyrus granule cells and cornus ammonis (CA) pyramidal neurons of the hippocampus. Representative images of Kgp staining: dog A 5.2‐yo, dog B 8.6‐yo, dog C 8.6‐yo, dog D 14.9‐yo. Scale bars 200 μm. (B) qPCR detection of *P. gulae* 16S DNA in frozen hippocampal tissue samples from the dogs shown in A. (C) Sequence analysis of the PCR product from dog D was performed and analyzed against the *P. gulae* sequence database (NCBI)

Lastly, we were interested in determining if *P. gulae* shared the same property of systemic translocation as *P. gingivalis*,^8^, ^26^, ^27^ especially to the brain,^1^ because if such an infection is present in the dog, the dog could become an important animal model for future COR388 studies in treating *P. gulae*/gingipain translocation from the oral cavity to the brain. To determine if dog brains are infected with *P. gulae*, postmortem brain tissue sections from the hippocampus of 4 dogs that were not part of the current COR388 PK/PD study reported here were stained for the presence of Kgp protein by IHC using a Kgp specific antibody. Kgp staining was detected in the hippocampus in the three older dogs (aged 8‐14 years), most prominently in pyramidal neurons in the cornu ammonis (CA), while little to no staining was seen in the 5‐year‐old dog (Figure 6A). Next, frozen hippocampal tissue samples from these same dogs were assessed by qPCR, with the results confirming the presence of *P. gulae* DNA (Figure 6B). Sequence analysis of the PCR product confirmed the identity of the qPCR product as *P. gulae* (Figure 6C). These results are consistent with a systemic infection of *P. gulae* in aged dogs and are concordant with the ability of this bacterium to translocate from the oral cavity and colonize other tissues including the brain.

## 4 DISCUSSION AND CONCLUSIONS

The results reported here support identification of a dose of COR388 necessary for inhibition of the gingipain virulence factor Kgp and the reduction of a naturally occurring oral *P. gulae* infection in dogs. These findings are consistent with our prior study demonstrating that targeting Kgp with COR388 decreased the bacterial load of *P. gingivalis* and AD related disease pathology in the brain of wild‐type mice orally infected with *P. gingivalis*.^1^ Importantly, we demonstrate here that Kgp proteases derived from *P. gulae* and *P. gingivalis* have similar proteolytic activity and that COR388 is a potent irreversible inhibitor of both proteases. Therefore, COR388 is predicted to have efficacy in diseases in which one or both of these bacterial pathogens are causative agents for disease pathology. In the pharmacokinetics and pharmacodynamics studies in the dog, COR388 was shown to be orally bioavailable and demonstrated dose‐ and time‐dependent Kgp active‐site target engagement, with good tissue distribution as evidenced by Kgp inhibition in both subgingival plaque and gingival crevicular fluid. These data also demonstrated that target engagement was sustained beyond peak plasma exposure, consistent with irreversible target binding. In addition to this long‐lasting nature of pharmacodynamic engagement on the target, Kgp inhibition at lower doses increased to that observed by a higher dose following prolonged administration. Specifically, the 0.5 mg kg^−1^ dose provided roughly 50% inhibition of baseline Kgp activity after a single dose but near complete inhibition after 28 days of dosing (Figure 4D). Re‐emergence of a low level of infection after 16 days following cessation of dose administration (Figure 3A) is consistent with the need for chronic repeated administration to suppress bacterial survival and replication. This sustained target inhibition decreased *P. gulae* load and downstream periodontal pathology with prolonged dosing. Lastly, we found evidence of *P. gulae* and neuronal Kgp in dog brains, similar to results recently published for *P. gingivalis* and Kgp in human AD brains.^1^

The focus of the current studies was to understand the pharmacokinetics and pharmacodynamic target engagement of COR388 in aged dogs, as such we did not power the study for cognitive effects of the test article which would have required a larger number of dogs, potentially a longer period of continuous dosing, and was outside of the scope of this study. Cognitive effects of COR388 administration, as well as changes in inflammatory and periodontal disease markers, are currently being studied in a large international trial of patients with mild‐to‐moderate AD (GAIN: ClinicalTrials.gov, NCT03823404).

We suggest that infection of the oral cavity is the most likely source of *P. gingivalis* or *P. gulae* and associated gingipains translocating to the brain and other organs in humans and dogs. The study reported here demonstrated that the small molecule gingipain inhibitor, COR388, has distribution to the oral cavity and oral biofluids, which contain a high bacterial burden, in sufficient concentrations to penetrate oral biofilms, engage the gingipain target, and reduce the oral bacterial load. The consistent reduction in Kgp activity coupled with the downstream reductions in bacterial load and RgpB activity in multiple independently isolated oral biofluids, as well as the buccal epithelial cells known to harbor intracellular infection, is evidence of broad systemic targeting of this pathogen by COR388. Specifically, the doses used in this study illustrate the therapeutic efficacy of COR388 in reducing a preexisting, established natural infection of the bacteria.

Because Kgp can be produced by both *P. gingivalis* as well as *P. gulae*, the target engagement of Kgp demonstrated here will reflect inhibition of the protease target in both species if both are present in the dogs. As we had previously demonstrated the efficacy of COR388 on Kgp and *P. gingivalis* bacterial infection in a mouse model, we chose to monitor *P. gulae* bacterial load in the current study to confirm that COR388 could also reduce the load of *P. gulae *in vivo.

Our previous study in mice demonstrated that COR388 is brain penetrant and efficacious in reducing established brain *P. gingivalis* load and AD pathology.^1^ This ability of COR388 to effectively inhibit gingipain with broad tissue distribution should promote the clearance of gingipains and *P. gingivalis*/*P. gulae* from multiple reservoirs in the body. We previously demonstrated that chronic administration of COR388 to *P. gingivalis* cultures in vitro did not lead to drug resistance,^1^ and no loss of bacterial suppression was seen in the current study when COR388 was administered continuously over a period of 90 days.

In conclusion, the Kgp active site is conserved between *P. gingivalis* and *P. gulae,* and COR388 shows potent inhibition of Kgp originating from both bacterial species. In the dog, systemic COR388 gained access to Kgp within oral biofilms that are normally difficult to penetrate, and Kgp target engagement reduced *P. gulae* load and downstream pathology, including decreased inflammation and periodontal disease. Since there is emerging evidence that *P. gulae* infects humans,^20^ and could potentially contribute to the infection in AD patients, it was important to demonstrate in this study that COR388 inhibits the critical virulence factor Kgp from both *P. gulae* and *P. gingivalis,* supporting ongoing use of COR388 to treat these infections in human clinical studies.

## 5 ETHIC STATEMENT

Aged Beagle dogs of both sexes ranging from 8 to 15 years of age, in generally good health and located in the Vivocore Inc dog colony, were utilized in this study. All procedures were reviewed and approved by Vivocore's Institutional Animal Care and Use Committee (IACUC) and were performed in accordance with the principles of the Animal for Research Act of Ontario and the guidelines of Canadian Council on Animal Care (CCAC).

## DISCLOSURES

Cortexyme funded this study. SSD and CL are co‐founders of Cortexyme, are employees of the company, and own Cortexyme stock. S.A‐K., MN, DR, FE, UH, and LJH are Cortexyme employees with stock options. MIR is a scientific advisor to Cortexyme and has options to purchase Cortexyme stock. SSD, and CL are inventors on two issued patents both entitled “Inhibitors of lysine gingipain” (US/9,975,473 and US/9.988,375) as well as two pending patent applications entitled “Inhibitors of arginine gingipain” (PCT/US2016/061197, filed on 9 November 2016) and “Ketone inhibitors of lysine gingipain” (PCT/US2017/051912, filed on 15 September 2017). SSD and CL are inventors on a patent application entitled “Methods of use for therapeutics targeting the pathogen P. gingivalis” (US2017/0014468, filed on 29 April 2015). LJH is an inventor on a patent application entitled “Gingipain activity probes” (62/459,456; filed on 15 February 2017). Cortexyme is the assignee of these patents and applications. The other authors declare that they have no competing interests.

## AUTHOR CONTRIBUTIONS

S.A‐K. designed and evaluated experiments and drafted the manuscript. MN grew *P. gulae* and *P. gingivalis* strains and performed Kgp and Rgp enzyme inhibition and activity assays. DR designed, performed, and analyzed PCR experiments of *P. gulae* DNA in dog oral biofluids and brain tissue and edited the manuscript. FE designed, performed, and analyzed immunohistochemcial experiments on dog brain tissues and helped draft the manuscript. UH helped analyze experiments. JA and ID helped design, coordinate, and conduct studies of COR388 in aged dogs, analyze experiments, and edit the manuscript. MIR evaluated experiments and edited the manuscript. SSD and CL helped develop and coordinate the project, design experiments, evaluate data, and draft the manuscript. LJH developed and coordinated the project, designed experiments, evaluated the data, and drafted the manuscript.

## Data Availability

The data that support the findings of this study are available from the corresponding author upon reasonable request.
