# Supplementary material for: Treatment of Porphyromonas gulae infection and downstream pathology in the aged dog by lysine‐gingipain inhibitor COR388
Source: Pharmacol Res Perspect. 2020 Jan 30;8(1):e00562. doi: 10.1002/prp2.562 (PMC6990966; doi:10.1002/prp2.562)

Figure 1C W83

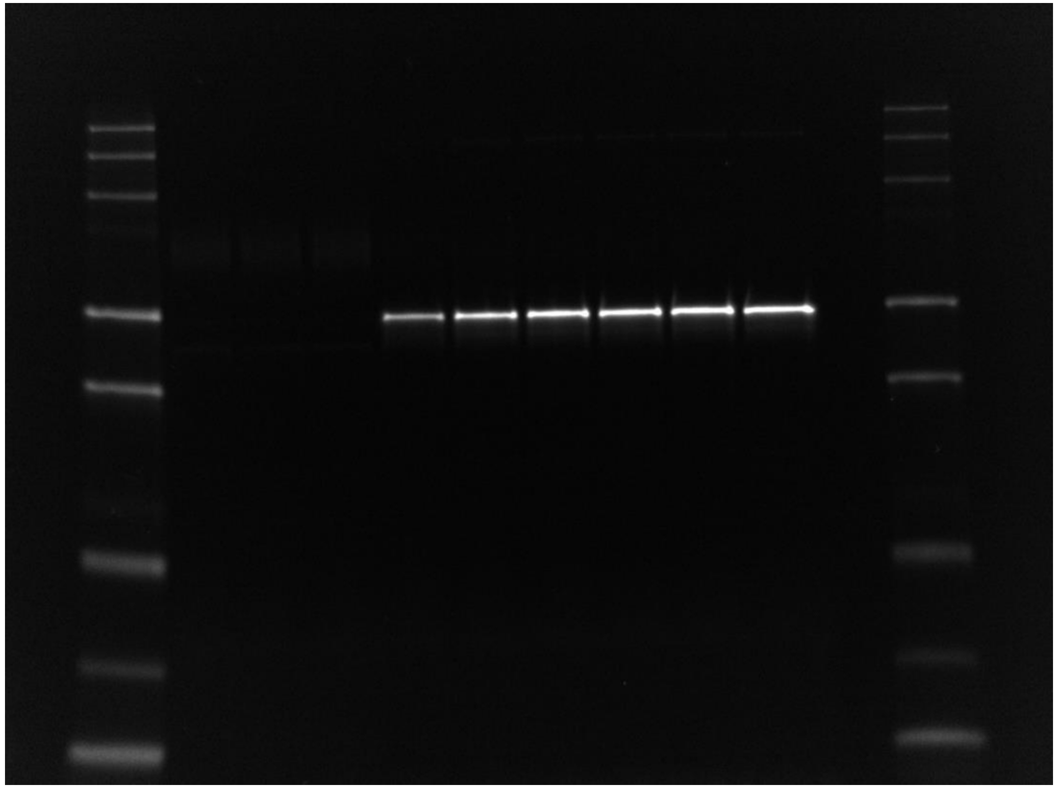

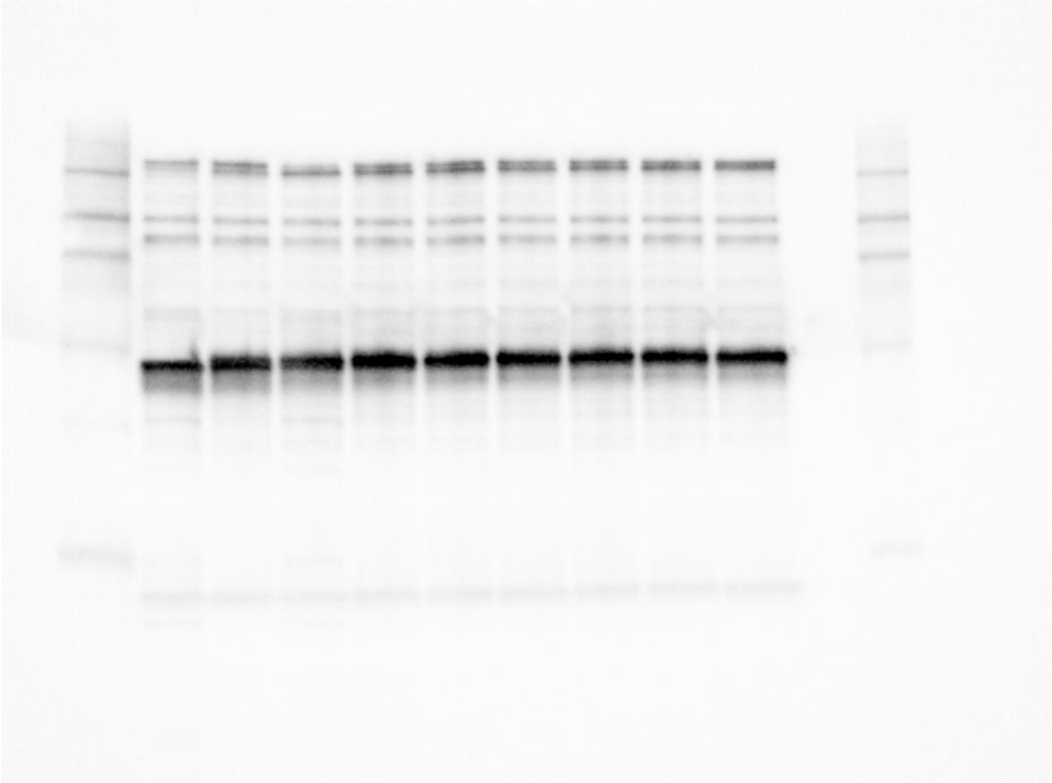

Figure 1C *P. gulae*

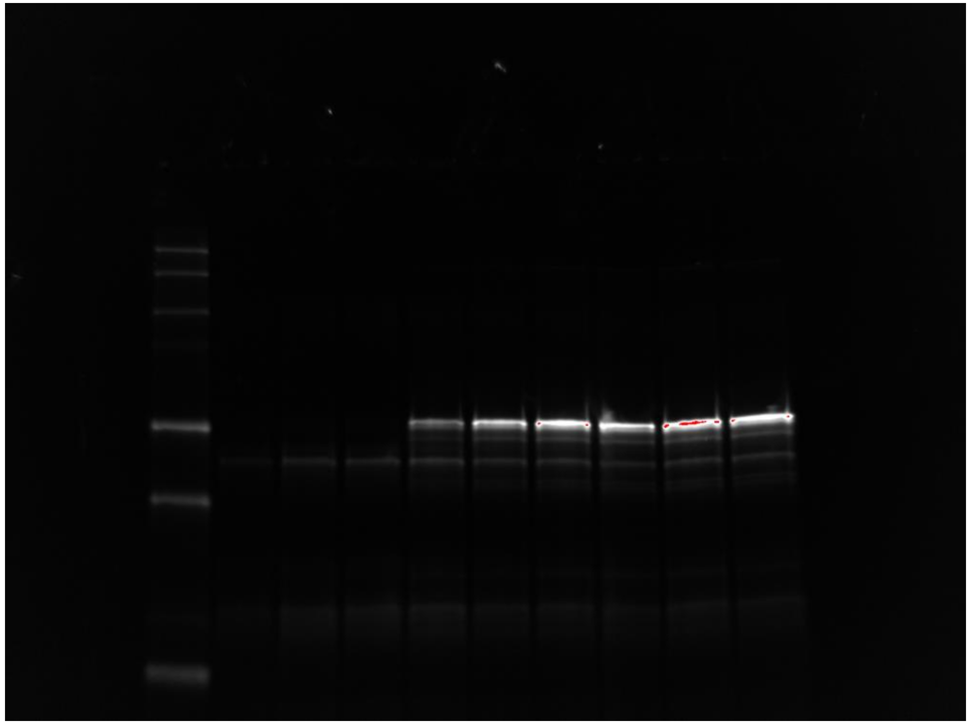

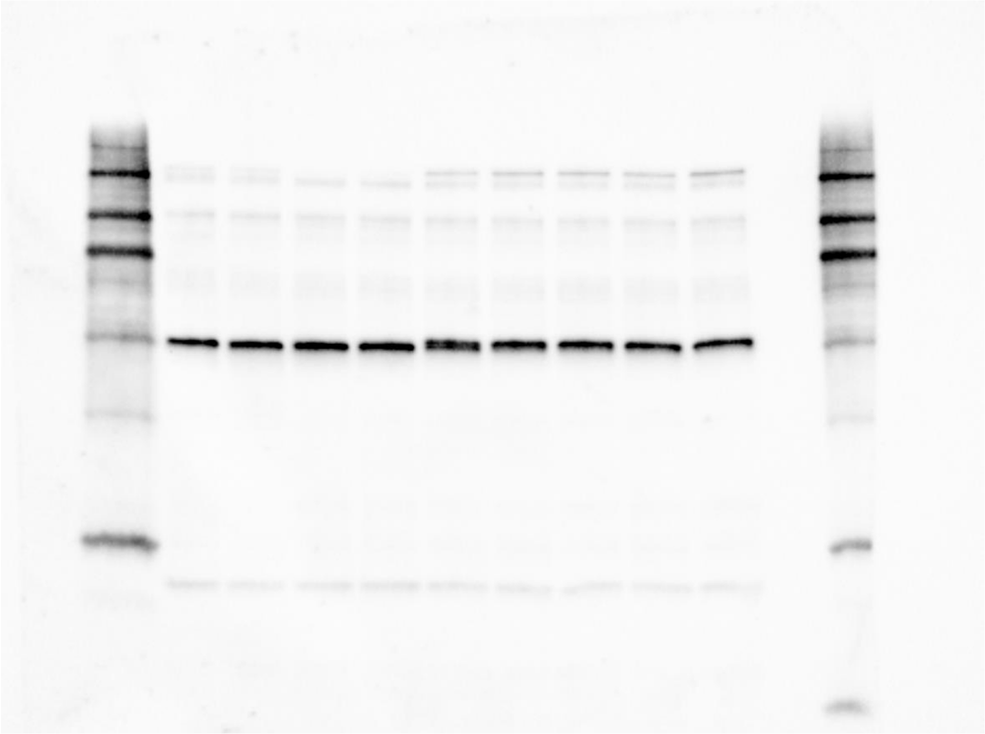

Figure 3C GCF – D02 study

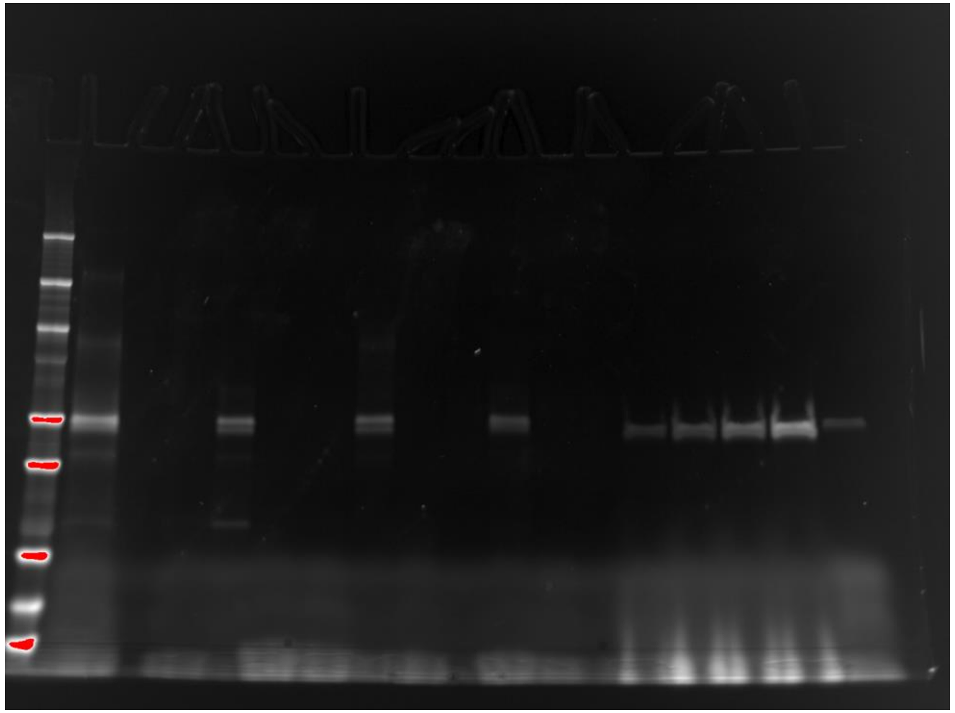

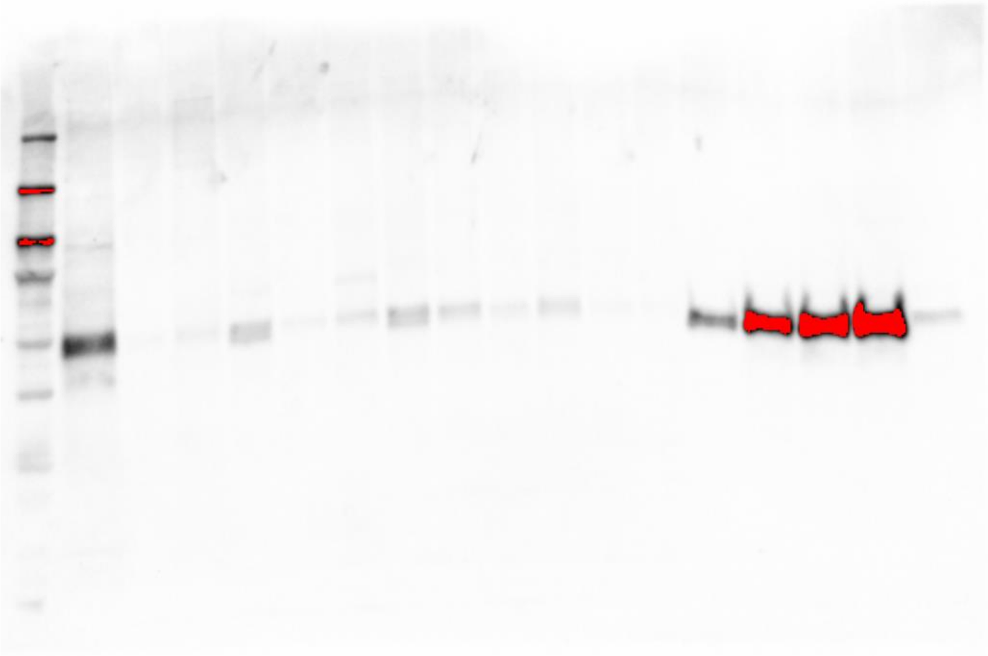

Figure 3C SGP - D02 study

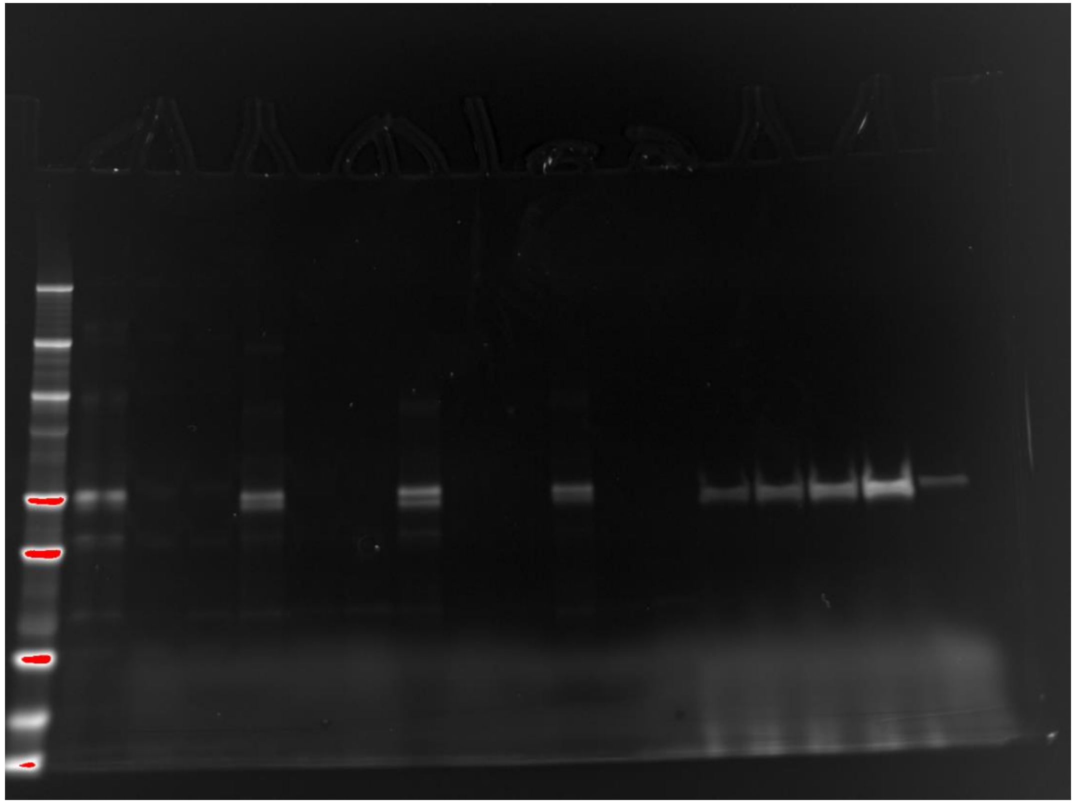

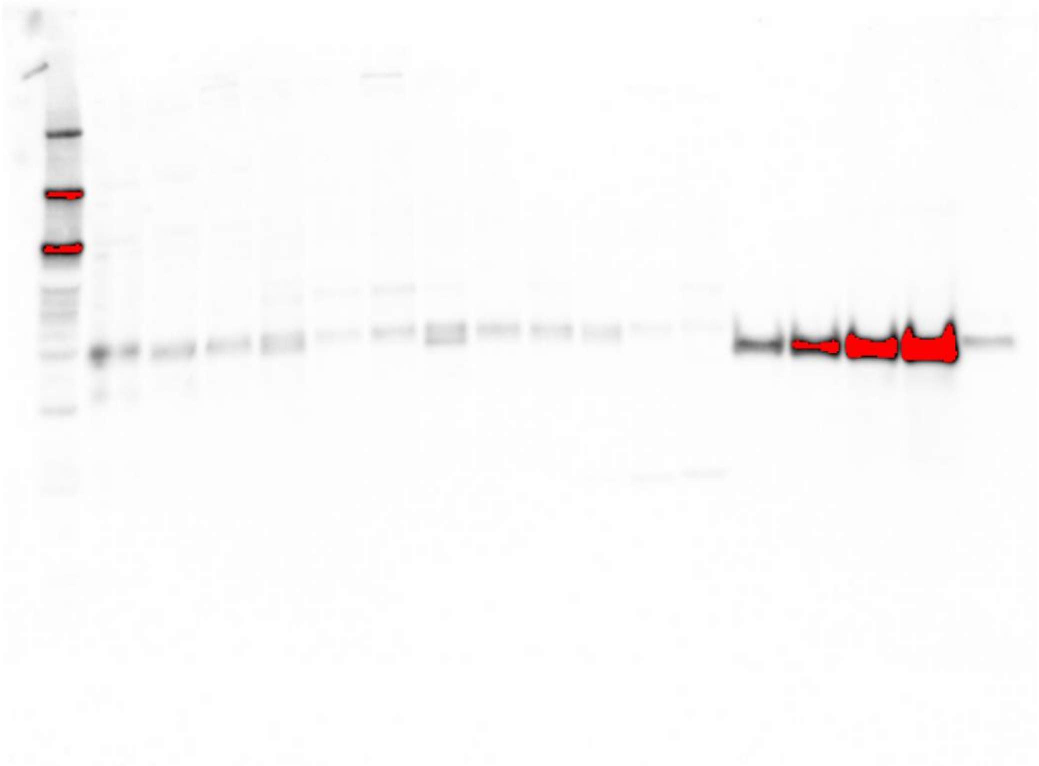

Supplemental Figure 1 A -- vehicle

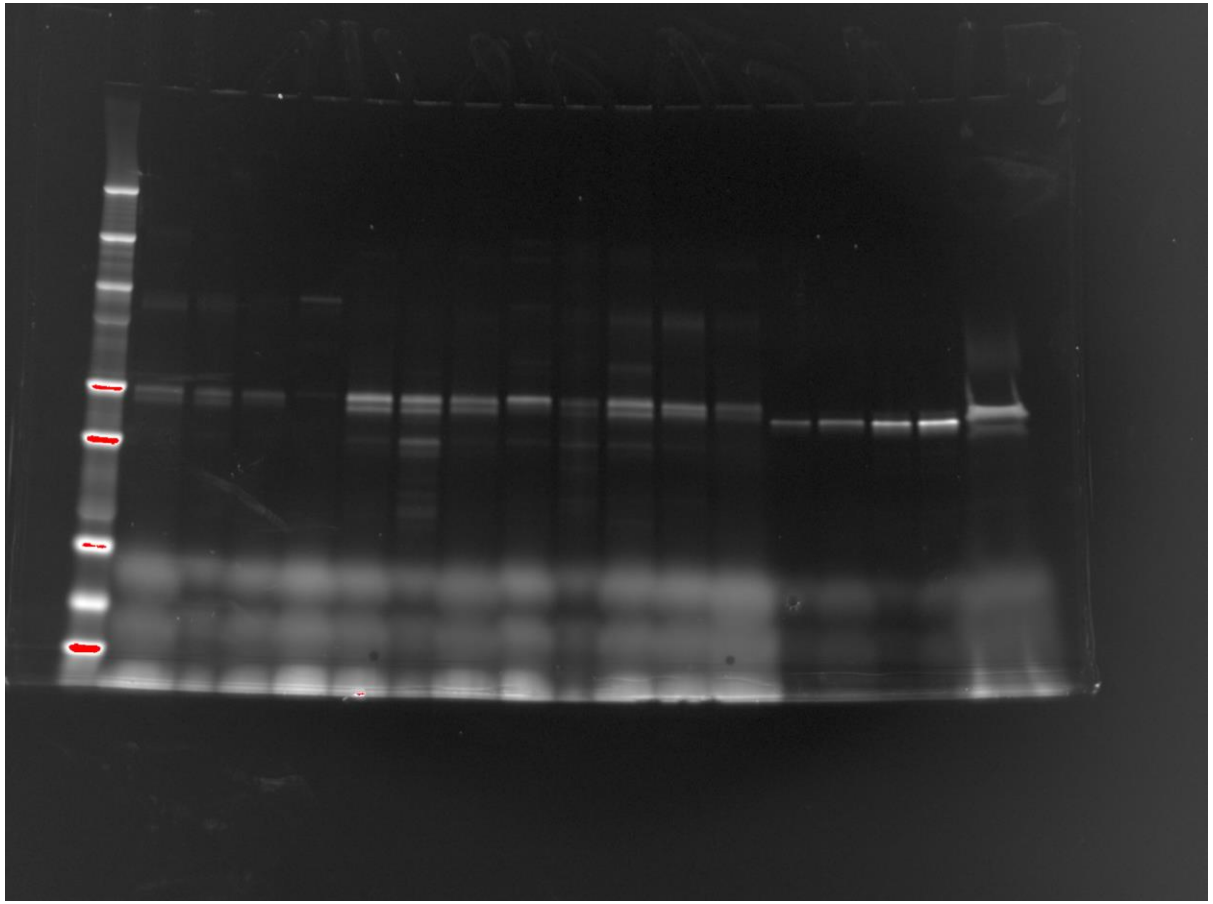

Supplemental Figure 1 B -- 0.15 mg kg^{-1}

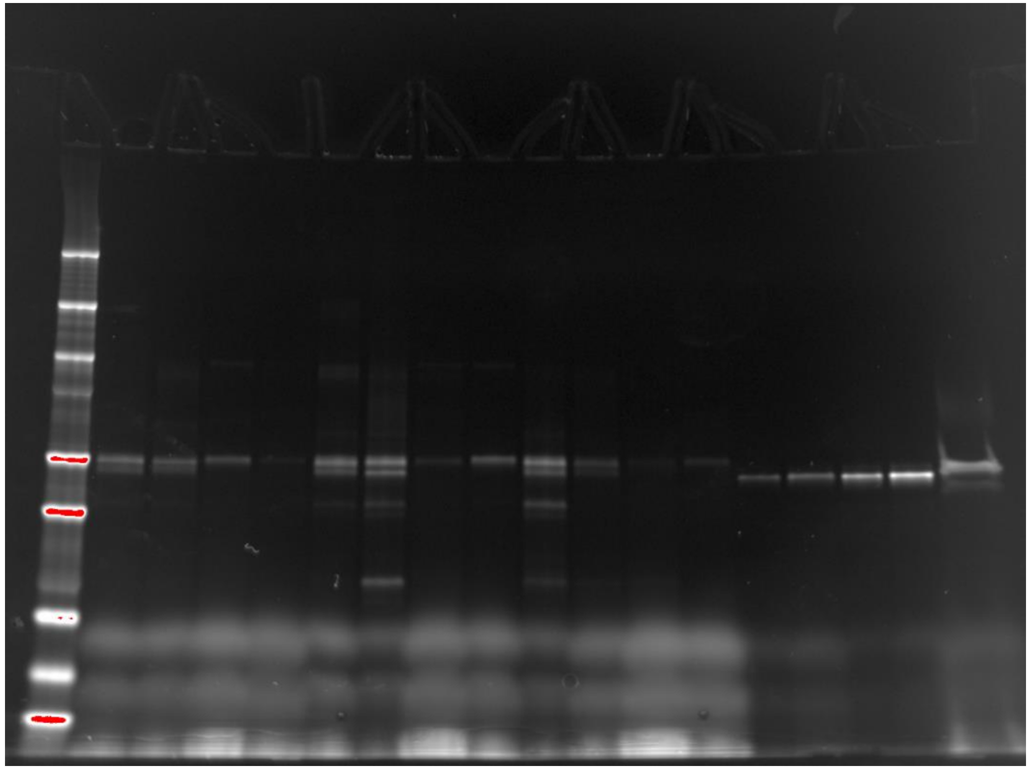

Supplemental Figure 1 C -- 0.5 mg kg^{-1}

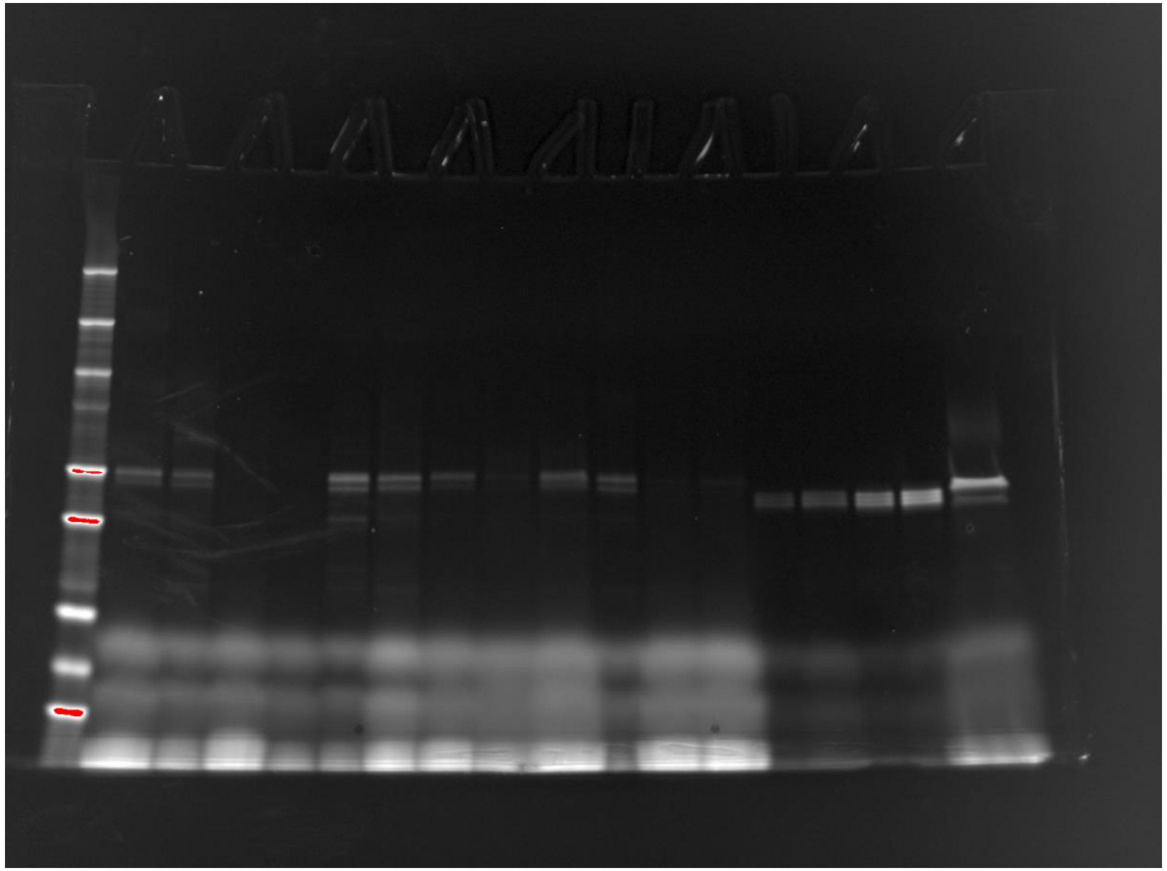

Supplemental Figure 1 D -- 1.5 mg kg^{-1}

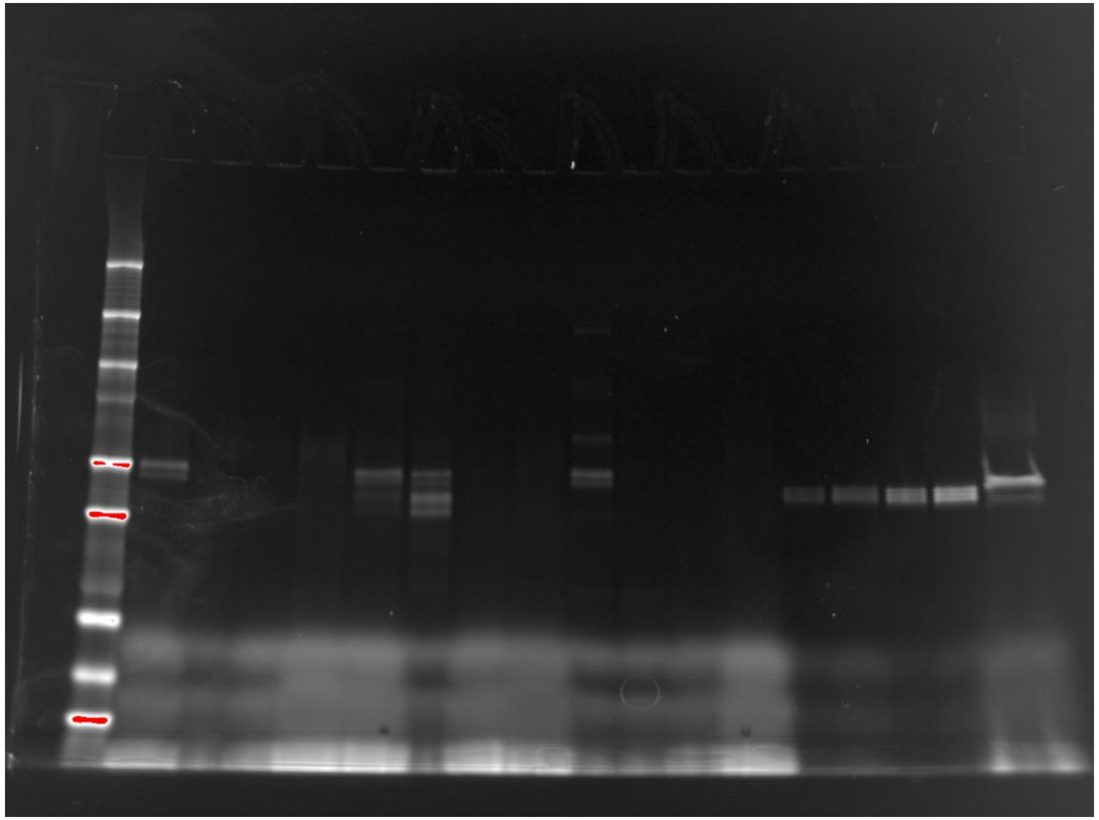

Supplemental Figure 1 E -- 12 hr

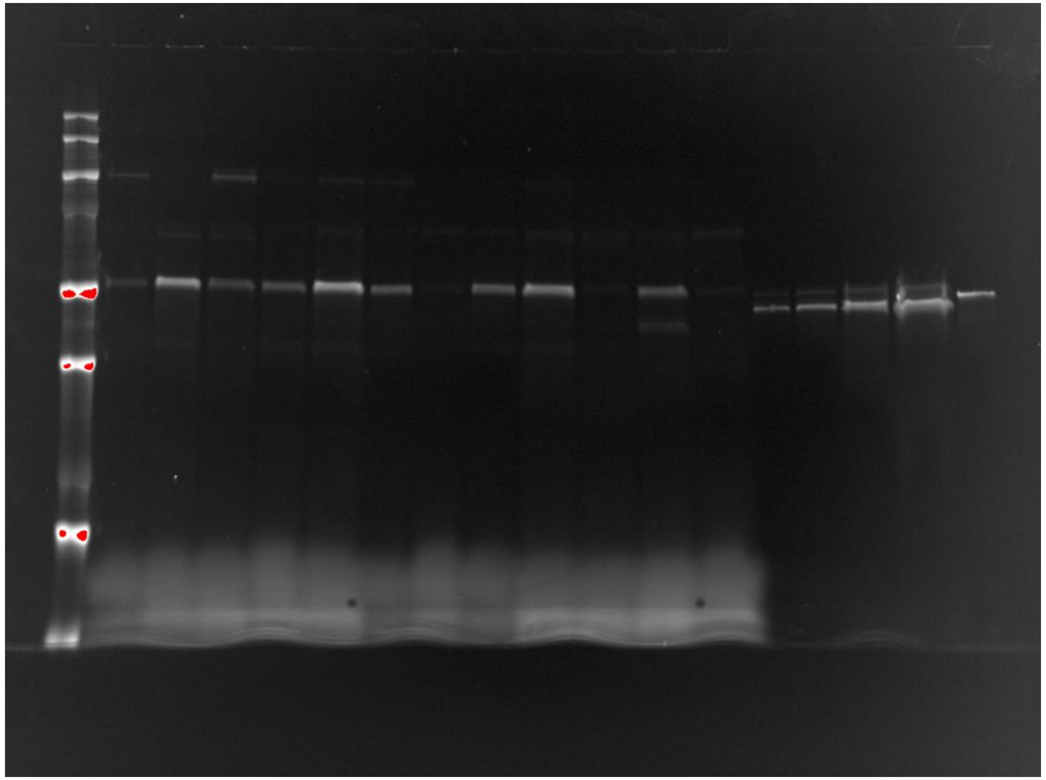

Figure 5B – day 90

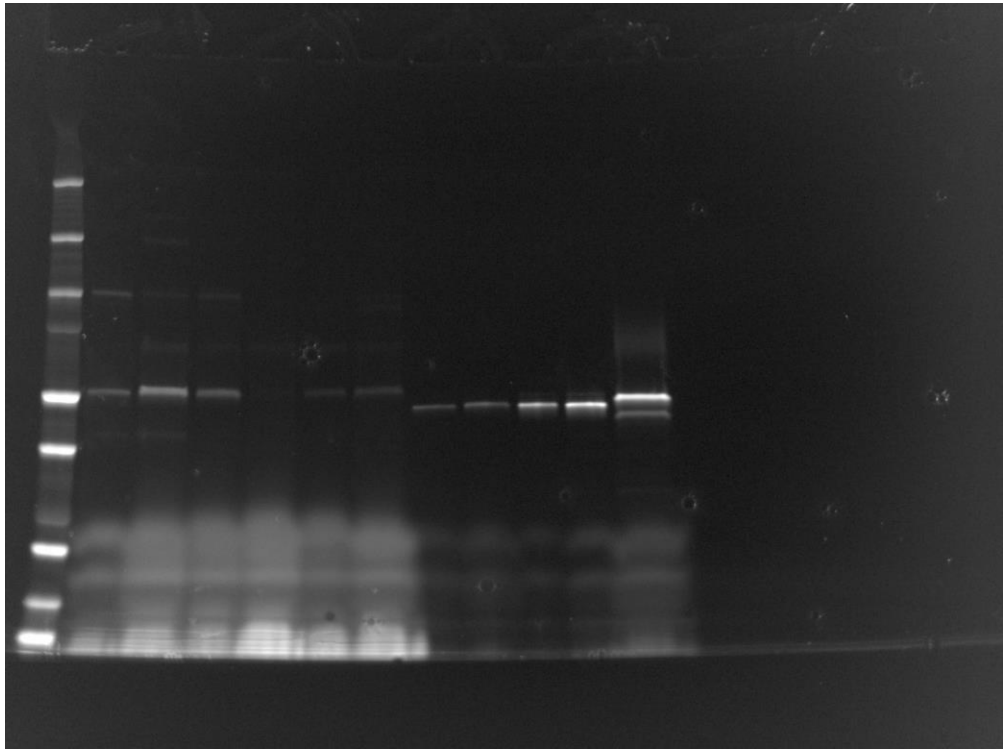

Original image for Figure 6A

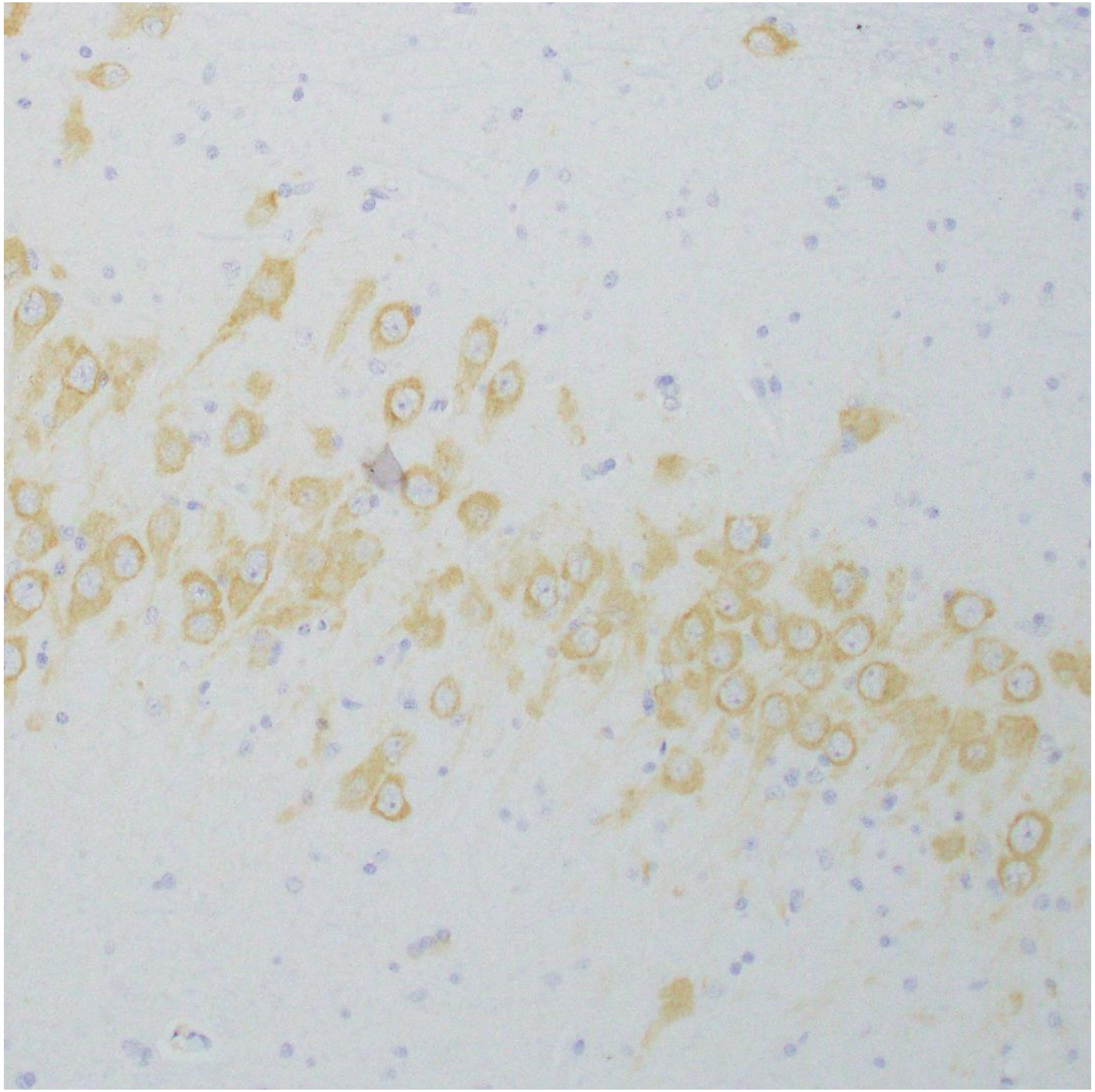

Original image for Figure 6B

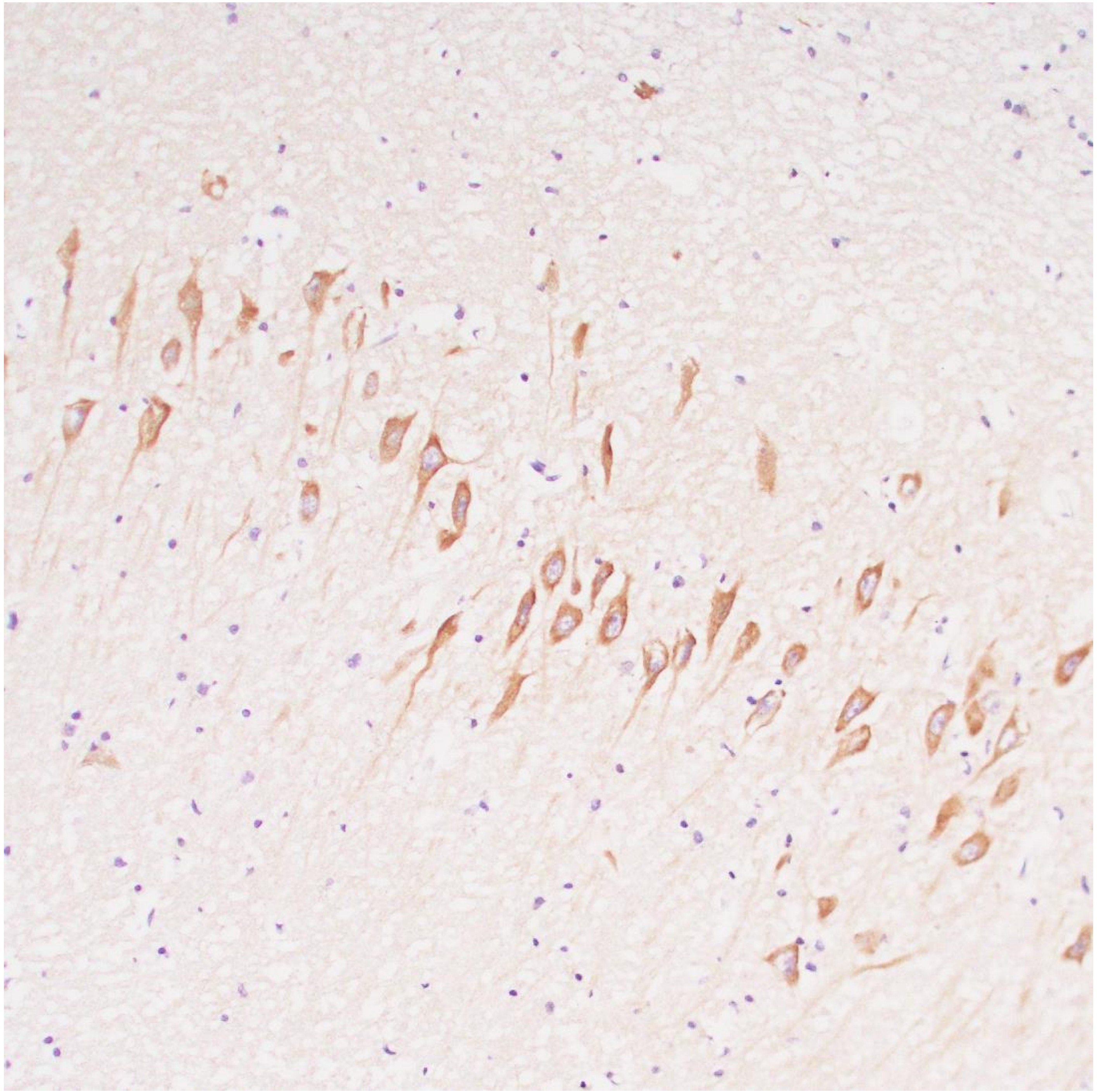

Figure 6C

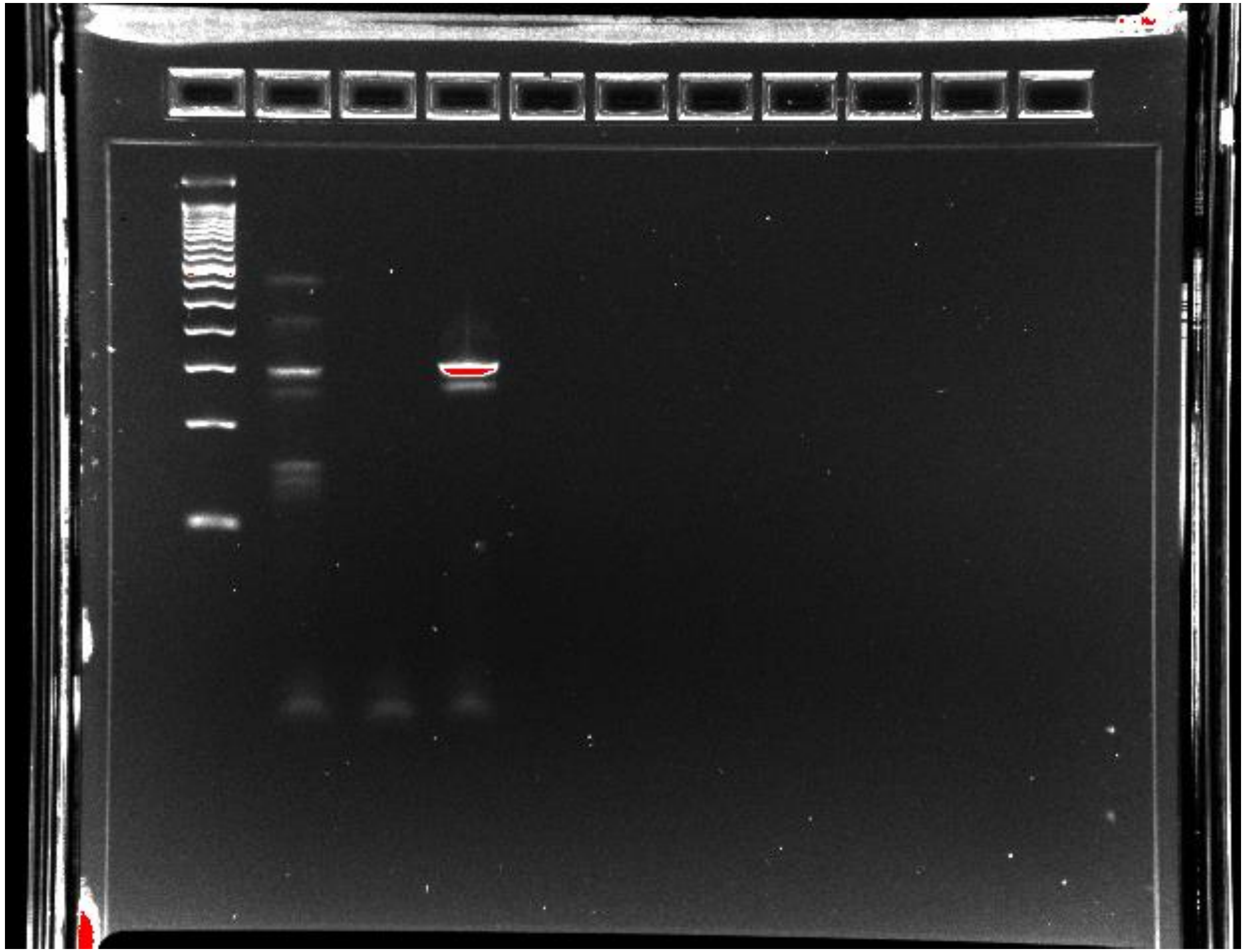

Supplement: Supplementary file 1 [file PRP2-8-e00562-s001.pdf]
