# Supplementary material for: Treatment of Porphyromonas gulae infection and downstream pathology in the aged dog by lysine‐gingipain inhibitor COR388
Source: Pharmacol Res Perspect. 2020 Jan 30;8(1):e00562. doi: 10.1002/prp2.562 (PMC6990966; doi:10.1002/prp2.562)
Supplement: Supplementary file 2 [file PRP2-8-e00562-s002.pdf]

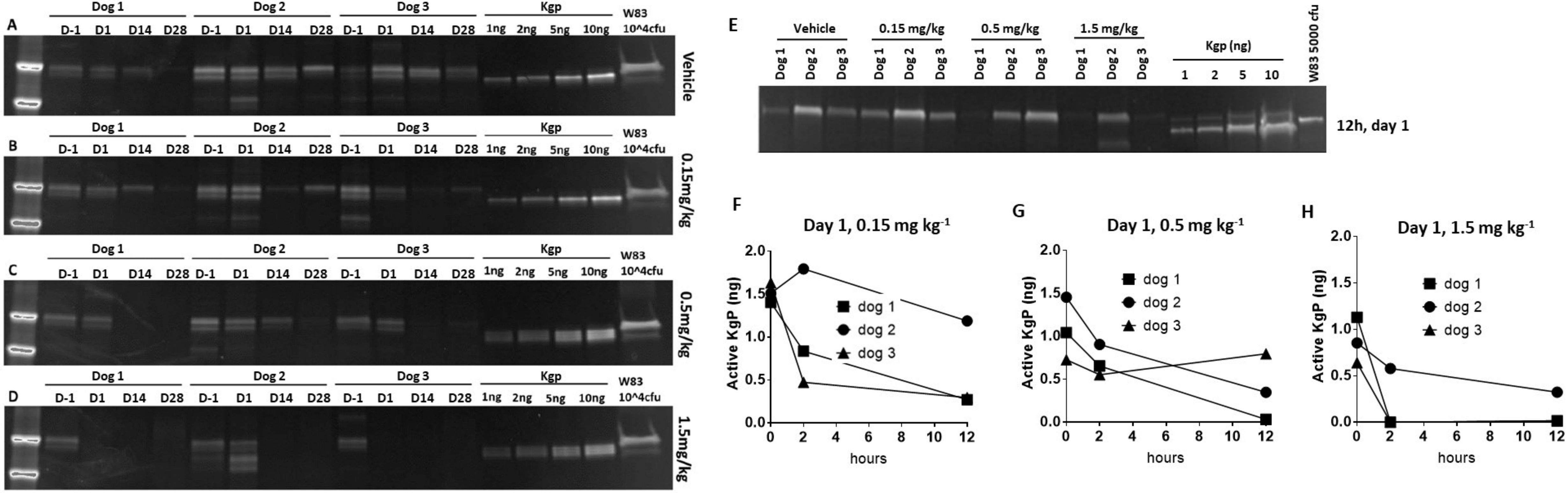

Supplemental Figure 1. Target engagement on the first day of dosing. A – D) COR553 probe detection of active Kgp in GCF samples. Day -1 baseline before dosing is shown along with samples from days 1, 14, and 28 of dosing. Day 1, 14, and 28 samples was taken 2h after dosing. Purified Kgp enzyme was used to generate a standard curve and a lysate of *P. gingivalis* W83 strain was also included as a standard control. E) COR553 probe detection of active Kgp in GCF on Day 1 at C_{trough} (12h post dose) were run separately from the other GCF samples. F, G, H) COR388 reduced Kgp specific activity (ng/mg of total GCF protein) in a dose and time-dependent manner. COR553 binding intensity from A – E were quantified and active Kgp determined using the Kgp standard curve. Active Kgp levels were normalized to the total amount of protein loaded as described in Methods.
